# Burden of hypereosinophilic syndromes in the United States: Patients’ perspective

**DOI:** 10.1016/j.jacig.2025.100501

**Published:** 2025-05-28

**Authors:** Jared Silver, Anna Kovalszki, Mary Jo Strobel, Dan Gratie, Amy G. Edgecomb, Cara Schmitt, Waseem Ahmed, Arijita Deb

**Affiliations:** a US Medical Affairs—Respiratory, GSK, Durham, NC; b University of Michigan, Ann Arbor, Mich; c American Partnership for Eosinophilic Disorders (APFED), Atlanta, Ga; d AESARA, Chapel Hill, NC; e Anti-Infectives and Respiratory, US Real-World Evidence and Health Outcomes Research, GSK, Collegeville, Pa; f Global Data Generation, GSK, London, United Kingdom; g Global Real-World Evidence & Health Outcomes Research, GSK, Upper Providence, Pa

**Keywords:** American Partnership for Eosinophilic Disorders, cross-sectional survey, health care resource utilization, hypereosinophilic syndromes, patient experience, quality of life, symptoms

## Abstract

**Background:**

Hypereosinophilic syndromes (HES) are rare hematologic disorders characterized by hypereosinophilia and eosinophil-driven organ damage/dysfunction. The HES diagnostic and treatment journey is poorly understood.

**Objective:**

We sought to describe the experience and disease burden of HES from a patient perspective.

**Methods:**

An online cross-sectional survey was completed by US patients aged 18 years and older with self-reported HES or caregivers (recruited via the American Partnership for Eosinophilic Disorders). Data on symptoms, diagnosis process, treatment, health care resource utilization, quality of life, and support structure were collected.

**Results:**

The mean age of the respondents (HES, n = 53; caregiver, n = 1) was 43.6 years (80% White and 57% male). One-quarter (26%) received their HES diagnosis in less than 3 months from first symptoms; 30% waited 3 months to 1 year, 37% 1 to 5 years, and 7% more than 5 years. Almost half of the respondents (n = 26) required hospital care 1 to 3 times in the 12 months before diagnosis. Most common symptoms were fatigue (96%), general discomfort (85%), wheezing (80%), rash (78%), and dry cough (76%). The most burdensome symptoms included leg swelling (100%), sweating (78%), and shortness of breath (64%). Symptoms associated with HES end-organ damage (respiratory and hypercoagulability symptoms) were observed. HES substantially impacted quality of life including work quality/productivity, finances, and relationships. Patients additionally wished for their doctor to show more empathy for their symptom burden, pain, and long-term mental health impacts.

**Conclusions:**

People with HES face long diagnostic journeys. The findings in this study highlight the heterogeneous symptoms, challenges, and multifactorial burden they experience, providing a voice for patients with HES and enhancing physician awareness to support improved diagnostics and management.

Hypereosinophilic syndromes (HES) are a group of rare hematologic disorders characterized by hypereosinophilia (>1500 cells/μL on ≥2 occasions for a minimum of 1 month) and eosinophil-driven organ damage and dysfunction not ascribable to secondary causes.^1^, ^2^, ^3^, ^4^, ^5^ The prevalence of HES is low (0.32-6.3 cases/100,000 people in the United States), and the disease is most commonly diagnosed between the ages of 20 and 50 years.^6^^,^^7^ HES presents with various clinical manifestations and, before the 2023 consensus on the classification of eosinophilic disorders,^8^ was commonly divided into the following subtypes: idiopathic HES (most common), myeloproliferative HES (M-HES), lymphocytic HES (L-HES), overlap HES, associated HES, and familial HES (rarest).^3^^,^^9^ HES symptoms occur across organ systems, evolve over time, and include fatigue, skin rashes, itching, shortness of breath, abdominal pain, muscle pain, and diarrhea, while comorbidities include asthma, congestive heart failure and valvular heart disease, stroke, and thrombus formation.^10^, ^11^, ^12^

HES identification and diagnosis are challenging because of the disease rarity, the varied clinical presentation, the lack of recognition, and overlapping characteristics with other eosinophilic-driven diseases.^4^^,^^5^^,^^9^^,^^12^ Patients with HES may experience delays of up to 20 years in receiving a diagnosis and effective targeted treatments.^13^^,^^14^ Poor disease control leads to an increase in oral corticosteroid (OCS) use, leading to significant adverse events,^13^^,^^15^^,^^16^ calling for an alternative approach to treat HES. Treatment choices for HES include immunosuppressants, cytotoxic therapies, and more targeted approaches such as injectable mAb medications.^4^^,^^9^ There are limited publications emphasizing the need for perspectives of patients with HES.^10^^,^^14^^,^^17^ Therefore, this study explored the disease burden of HES to facilitate the understanding of unmet patient needs and direct improvements in diagnosis and management.

This study aimed to describe the experience of patients with HES from initial reported symptoms, through diagnosis, to health care resource utilization (HCRU), quality-of-life (QoL) burdens, support structure, and treatment experiences. It was performed in conjunction with a patient advocacy group, the American Partnership for Eosinophilic Disorders (APFED). APFED is a nonprofit organization founded in 2001 that aims to improve the lives of individuals with eosinophil-associated diseases through education and awareness, research, support, and advocacy.^18^ Organizations such as APFED contribute to patients’ understanding of diseases, identify unmet needs and strategies to address them, and enable patient data collection, all of which can provide invaluable insights to health care providers (HCPs) and researchers.

## Methods

### Study design

This was a noninterventional online cross-sectional survey using multiple-choice and free-response questions to collect data on symptom burden, the diagnostic process, treatment history, HCRU, support structure, and overall disease experience of patients with self-reported HES or their caregivers. A study database was created and the survey was piloted by patient volunteers before the survey launch to ensure readability and functionality. These volunteers were eligible to participate in the final survey, which is provided in Table E1 (in the Online Repository available at www.jaci-global.org). On joining APFED, prospective members indicate their interest in certain eosinophilic disorders and subsequently receive targeted communications on the basis of said interests (no diagnoses are collected).^19^ Thus, potential respondents who had indicated HES as a disease of interest were identified by APFED and contacted for participation. All survey data collection and analysis were performed on an anonymous basis. The survey was open from February 28, 2022, to May 5, 2022. This study includes data from the US population.

### Eligibility criteria

The survey was completed only by patients with HES or their caregivers. Eligible patients were 18 years or older, had a self-reported HES diagnosis, and were able to provide electronic informed consent and complete the online survey. The participants did not provide medical records. Caregivers, who were 18 years or older, provided a proxy for adolescents, children, and those patients who were unable to complete the survey independently. Exclusion criteria included failure to meet the inclusion criteria or unwillingness to participate in the survey or complete the informed consent form. Respondents who expressed an interest in the survey completed a brief screening questionnaire to ensure that the study inclusion and exclusion criteria were met.

### End points and assessments

Respondents completed a one-time, electronic, cross-sectional, 104-item survey (Table E1) and received a $50 honorarium on completion. Demographic information included age, gender, ethnicity, US state of residence, insurance status, education level, employment status, and household income (items 3-12). QoL information included questions on the impact of HES on daily life, work/school, recreation, social life and relationships, outings, and other areas (items 81-96). Diagnosis and treatment history included age at HES diagnosis, disease subtype (pre-2023 classification^3^^,^^9^), comorbid conditions, specialization of diagnosing HCP, family history of HES, detailed diagnosis history, treatment history, treatment satisfaction and impact, and reason for any treatment discontinuation (items 13-34).

As part of the assessment of treatment history, the survey explored the intensity and duration of HES symptoms in the following areas: general, skin, sinus/nasal/lung, neurologic, muscular/joint, gastrointestinal (GI), and cardiovascular (items 35-80). The respondents’ support structure and sources of information regarding HES were assessed by identifying who they could talk to about their condition and who they were able to approach to access HES information (items 97-99). HCRU was assessed according to the frequency of visits over 12 months for urgent care, emergency room, hospital admission, office visits, specialist visits, and all-cause medication use (items 100-103).

### Sample size and statistical analysis

This was an exploratory study using a convenience sample; thus, sample size and power were not calculated. The survey was sent to APFED’s membership, which included approximately 2100 people, of whom 266 had voluntarily identified as having an HES diagnosis. In addition, the survey was promoted to 247 individuals in the Eosinophil.Connect Patient Insights Network^SM^ (hosted on the Invitae platform),^20^ who self-identified as having HES. Only descriptive analyses were used when interpreting study results. Data collected through free-text answers were qualitatively summarized to capture broad themes and sentiments.

### Ethics

Appropriate ethical approval was obtained via a central institutional review board (WCG, Puyallup, Wash) before any participant recruitment. The survey included an electronic informed consent form.

### Data availability

For requests for access to anonymized subject-level data, please contact the corresponding author.

## Results

### Demographic and clinical characteristics

There were 64 survey responses. Five surveys were not included because of being incomplete (n = 3) or lost to follow-up (n = 2), and so the overall analysis included 59 surveys. Here, we report data from US respondents (N = 54 [53 people with a self-reported HES diagnosis and 1 caregiver]; see Table E2 in this article’s Online Repository at www.jaci-global.org).

Most respondents (59%) were aged 18 to 44 years, with a mean age of 43.6 years (Table E2). Most respondents were White (80%) and male (57%). In total, 21 of 54 (39%) respondents were in full-time employment, and Medicare was the most frequent health care coverage (41%). Respondents were mostly located in the southern, western, and northeast US states (26%-32%), with a lower proportion in the Midwest (13%).

#### HES diagnosis

**Fig 1:**
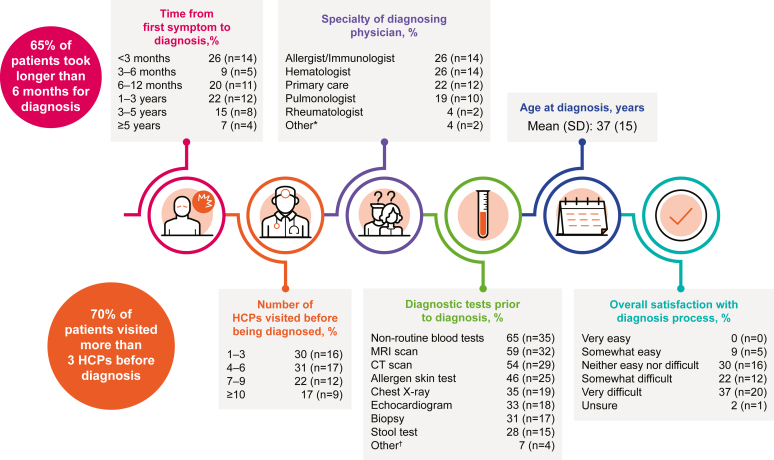
People with HES experience a long and complicated route to diagnosis. Results from items 13, 17, 18, 20, 21, and 22 of the survey instrument (Table E1). *CT,* Computerized tomography; *MRI*, magnetic resonance imaging. Originally presented at the American College of Allergy, Asthma and Immunology 2022 Annual Scientific Meeting.^21^ ∗Includes cardiologist, gastroenterologist, and neurologist. †Includes spinal tap, endoscopy, colonoscopy, psychological tests, and pulmonary function tests.

Most respondents (59%) found obtaining an HES diagnosis “somewhat” or “very” difficult (Fig 1; see also Table E3 in this article’s Online Repository at www.jaci-global.org). In total, 26% received their HES diagnosis within 3 months of their first symptoms, 30% waited between 3 months and 1 year, 37% waited 1 to 5 years, and 7% waited more than 5 years. Seventy percent of respondents visited more than 3 HCPs before receiving their diagnosis, with 17% visiting more than 10 HCPs. The mean age of receiving an HES diagnosis was 37 years. Most respondents had idiopathic HES (59%), followed by M-HES (28%) and L-HES (6%) (see Table E4 in this article’s Online Repository at www.jaci-global.org). More than half (52%) had no family history of HES, and 20% were unaware of having a family history of HES.

#### Comorbidities

The top 5 comorbidities associated with HES were asthma (54%), anxiety (26%), chronic skin disease (24%), GI disorders (22%), and chronic obstructive pulmonary disease, emphysema, or chronic bronchitis (15%) (Table E4). The most common eosinophilic comorbidities were respiratory (asthma) and GI (esophagitis and gastritis/gastroenteritis) disorders (see Table E5 in this article’s Online Repository at www.jaci-global.org). Respondents with HES reported comorbid conditions that could result in end-organ damage, most commonly eosinophilic asthma (37%), eosinophilic esophagitis (31%), eosinophilic gastritis/gastroenteritis (20%), and deep vein thrombosis (13%). A codiagnosis of eosinophilic granulomatosis with polyangiitis was reported by 13% of respondents.

### Symptoms, complications, and HES end-organ damage

#### HES symptoms

**Fig 2:**
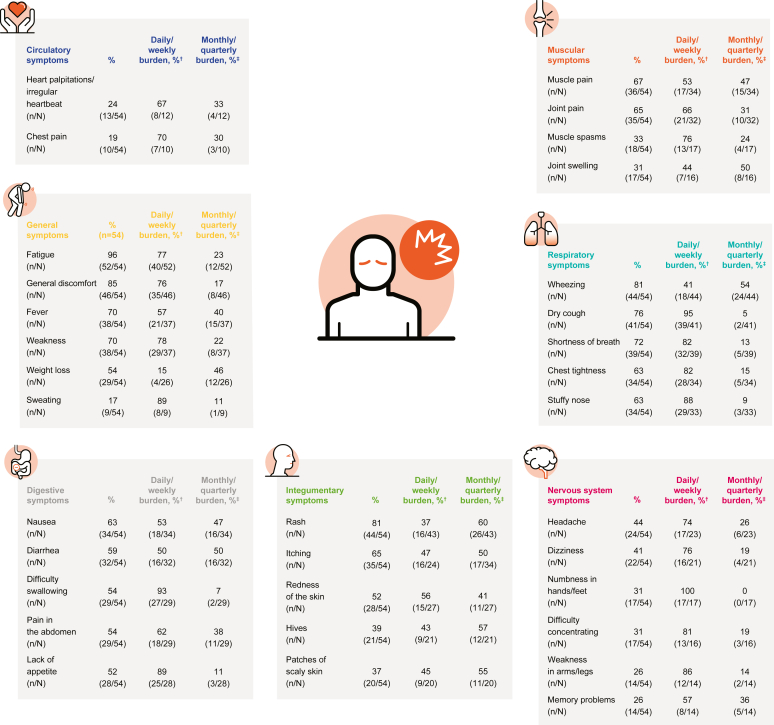
Common symptoms∗ experienced by patients with HES. Results from items 35 and 37 (general); 39 and 41 (integumentary); 43 and 45 (respiratory); 56 and 58 (nervous system); 62 and 64 (muscular); 66 and 68 (digestive); and 72 and 74 (circulatory) of the survey instrument (Table E1). *NA*, Not applicable. ∗Where possible, the 5 most common symptoms per category are presented (assessed in items 35, 39, 43, 62, 66, and 72). Symptoms may have been reported in ≥1 category (eg, shortness of breath captured in items 43 [respiratory; 72%] and 72 [circulatory; 22%]). †Reported experiencing symptoms daily/weekly in items 37, 41, 45, 58, 64, 68, and 74 (N = responders to these items, differs from the responders to items 35, 39, 43, 56, 62, 66, and 72, respectively). ‡Reported experiencing symptoms monthly/quarterly in items 37, 41, 45, 58, 64, 68, and 74 (N = responders to these items, differs from the responders to items 35, 39, 43, 56, 62, 66, and 72, respectively). Other responses: first-time experience; NA or prefer not to answer.

Respondents with HES experienced a wide range of symptoms affecting the respiratory, nervous, GI, circulatory, dermatological, and muscular systems as well as general symptoms such as fatigue and discomfort. The most common symptoms were fatigue (96%), general discomfort (85%), rash (81%), wheezing (81%), and dry cough (76%) (Fig 2; see also Table E6 in this article’s Online Repository at www.jaci-global.org).

**Fig 3:**
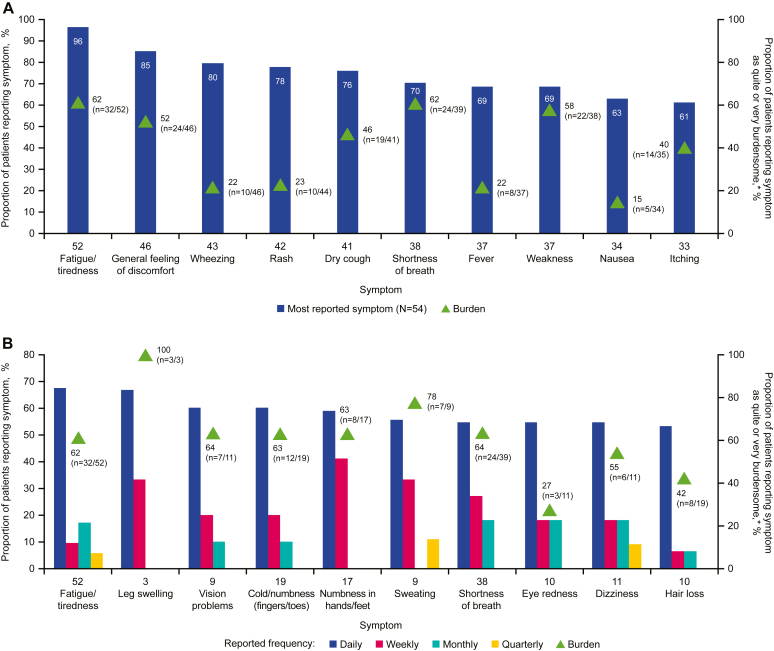
A, The top 10 most reported symptoms (any frequency) and their associated burden. **B,** The 10 symptoms that were most likely to be experienced daily and their burden. *NA*, Not applicable. The “n” along the x axes corresponds to the respondents who reported daily, weekly, monthly, or quarterly frequencies (“NA or prefer not to answer” were excluded) for the selected symptoms in items 37, 41, 45, 58, 64, 68, and 74 of the survey instrument (Table E1; may differ from responders for the same symptoms in items 35, 39, 43, 56, 62, 66, and 72 presented in Fig 2); the “n” next to the triangle markers corresponds to the combined number of respondents who rated the overall burden for said symptoms to be “quite a bit” or “very much” in items 36, 40, 44, 57, 63, 67, or 73 of the survey instrument out of the total number of respondents to the same item for the same symptom. One patient reported the symptom “Other” with the frequency “daily”; this includes fainting, excessive sleep, mixed axonal and generalized demyelinating, sensorimotor polyneuropathy, jerky movements, and tingling sensation in fingers and toes.

The most commonly experienced symptoms, which were also considered burdensome, included fatigue, general discomfort, wheezing, rash, dry cough, shortness of breath, fever, weakness, nausea, and itching (Fig 3, *A*). The symptoms that most respondents reported experiencing daily included fatigue, leg swelling, vision problems, cold/numbness in fingers and toes, numbness in hands/feet, sweating, shortness of breath, eye redness, dizziness, and hair loss; with the exceptions of hair loss and eye redness, at least 54% respondents rated these symptoms as “quite” or “very” burdensome (Fig 3, *B*).

### Treatment history

In terms of treatments reported before HES diagnosis, steroids (52%; prednisone/methylprednisolone, administration route not specified), injectable mAb treatments (28%; mepolizumab/alemtuzumab/benralizumab), nonprescription management such as over-the-counter treatments (22%), and hydroxyurea (17%) were most commonly reported (indications for these previous medications not captured; Table I). Half of the respondents (n = 27) required hospital admission, 1 to 3 times (n = 26) or 7 to 9 times (n = 1) in the 12 months before HES diagnosis (see Table E7 in this article’s Online Repository at www.jaci-global.org). Nine respondents (17%) visited the emergency room 1 to 3 times (n = 7), 4 to 6 times (n = 1), or 7 to 9 times (n = 1) in the 12 months before HES diagnosis.

**Table I:** HES treatments: Previous and current treatments and reasons for discontinuation

HES treatment∗ (N = 54)	Previous treatment, n (%)		Current HES treatment, n (%)			Total no. of patients who discontinued, N† (%)	Reason for discontinuation, n (% of N†)				
	Treatments before HES diagnosis (past 5 y)	Previous HES treatments	All (N = 54)	I-HES (N = 32)	M-HES (N = 15)		HCP chose to stop	Health insurance no longer covered	Affordability	No longer receiving benefit	Other‡
Steroids (prednisone or methylprednisolone)§	28 (52)	30 (56)	12 (22)	8 (25)	2 (13)	27 (50)	17 (63)	1 (4)	0 (0)	4 (15)	5 (19)
Hydroxyurea	9 (17)	18 (33)	13 (24)	10 (31)	2 (13)	15 (28)	1 (7)	3 (20)	5 (33)	4 (27)	2 (13)
Chlorambucil	4 (7)	15 (28)	5 (9)	3 (9)	2 (13)	13 (24)	2 (15)	2 (15)	5 (38)	3 (23)	1 (8)
Vincristine	8 (15)	12 (22)	0 (0)	0 (0)	0 (0)	12 (22)	6 (50)	0 (0)	0 (0)	0 (0)	6 (50)
mAb injectable medications‖	15 (28)	10 (19)	19 (35)	12 (38)	2 (13)	8 (15)	2 (25)	0 (0)	0 (0)	1 (13)	6 (63)
Methotrexate	1 (2)	7 (13)	3 (6)	1 (3)	2 (13)	6 (11)	1 (17)	1 (17)	0 (0)	3 (50)	1 (17)
Nonprescription management¶	12 (22)	6 (11)	9 (17)	0 (0)	0 (0)	6 (11)	2 (33)	0 (0)	1 (17)	1 (17)	2 (33)
IFN-α	NR	6 (11)	0 (0)	0 (0)	0 (0)	6 (11)	3 (50)	0 (0)	1 (17)	1 (17)	1 (17)
Cyclosporine	1 (2)	3 (6)	8 (15)	0 (0)	8 (53)	3 (6)	1 (33)	1 (33)	0 (0)	1 (33)	0 (0)
Ivermectin	2 (4)	3 (6)	1 (2)	0 (0)	0 (0)	3 (6)	0 (0)	1 (33)	0 (0)	2 (67)	0 (0)
Tyrosine kinase inhibitors	2 (4)	3 (6)	2 (4)	0 (0)	1 (7)	3 (6)	0 (0)	1 (33)	0 (0)	2 (67)	0 (0)
Investigational product	NR	0 (0)	0 (0)	0 (0)	0 (0)	0	0 (0)	0 (0)	0 (0)	0 (0)	0 (0)
Azathioprine	0 (0)	0 (0)	1 (2)	0 (0)	0 (0)	0 (0)	0 (0)	0 (0)	0 (0)	0 (0)	0 (0)
None#	3 (6)	0 (0)	0 (0)	0 (0)	0 (0)	NR	NR	NR	NR	NR	NR
Others∗∗	2 (4)	0 (0)	0 (0)	0 (0)	0 (0)	NR	NR	NR	NR	NR	NR
Unsure	NR	0 (0)	1 (2)	0 (0)	0 (0)	NR	NR	NR	NR	NR	NR

Responses to items 14, 24, 27, and 33 of the survey instruments (Table E1). Data on current HES treatments include an analysis of the overlap between items 14 and 27.

*I-HES*, Idiopathic hypereosinophilic syndrome; *NR*, not reported.

∗ Nonmutually exclusive groups.

† Total respondents per question.

‡ “I chose to stop for other reasons”: the question did not ask respondents to elaborate on what the other reasons were.

§ Administration route not specified.

‖ Includes mepolizumab, alemtuzumab, and benralizumab.

¶ Includes over-the-counter medications: Tylenol and Flonase nasal spray.

# One patient indicated that the reason for not receiving any treatment before HES diagnosis was that the HCP did not prescribe any treatments. All others did not report reasons for not receiving treatment.

∗∗ Includes GI medications (unspecified), etoposide 50-mg tablet, albuterol (unspecified dosage form), montelukast tablet, and cromolyn (unspecified dosage form).

**Fig 4:**
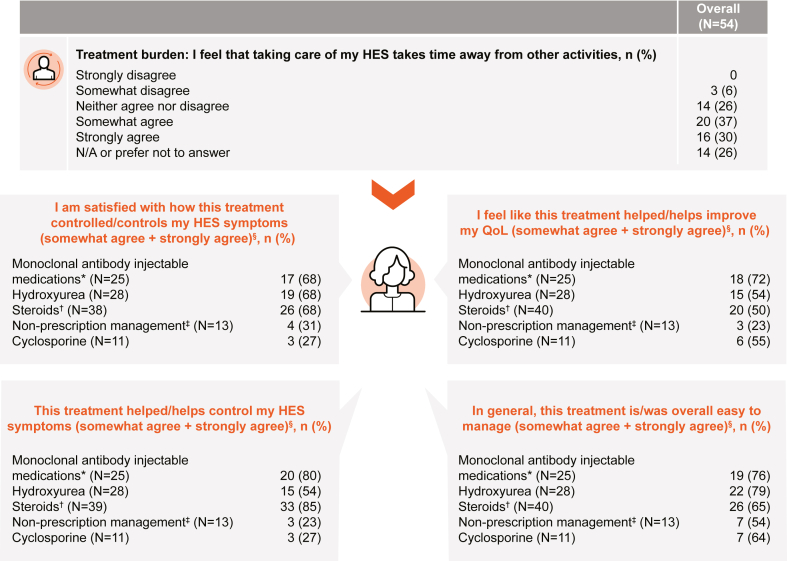
Treatment satisfaction. Results from items 29, 30, 31, 32, and 34 of the survey instrument (Table E1). *NA*, Not applicable. ∗Includes mepolizumab, alemtuzumab, and benralizumab. †Prednisone/methylprednisolone, administration route not specified. ‡Includes over-the-counter medications: Tylenol and Flonase nasal spray. §Data shown for the 5 treatment options that received the highest proportion of combined “somewhat agree” and “strongly agree” replies in items 29, 30, 31, and 32 of the survey instrument out of the total number of respondents (N) who replied to each item, respectively.

Most respondents (78%) reported receiving HES treatment within 6 months of diagnosis; however, a minority (11%) experienced delays of between 1 and 5 years, and 1 (2%) respondent reported a delay of more than 10 years (see Table E8 in this article’s Online Repository at www.jaci-global.org). Injectable mAb treatments were the most prescribed current treatments for HES, with 35% of respondents currently receiving them (Fig 4; Table I). The other most common current treatments were hydroxyurea (24%) and steroids (22%). Of the 32 patients with idiopathic HES, injectable mAbs were the most common current treatment (38%), followed by hydroxyurea (31%) and steroids (25%). Most (53%) of the 15 patients with M-HES were being treated with cyclosporine at the time of the survey (Table I).

Steroids were the most discontinued treatment (50%), followed by hydroxyurea (28%), chlorambucil (24%), and vincristine (22%). HCP decision was the most common reason for treatment discontinuation, accounting for 63% of steroid discontinuations and 50% of both vincristine and IFN-α discontinuations. Withdrawal of health insurance coverage, affordability, loss of treatment benefit, and other reasons were also cited (Table I).

### Patient experience and QoL impact

Patients reported a substantial treatment burden associated with HES, with 67% “somewhat” or “strongly” agreeing that caring for their HES took time away from other activities (Fig 4; see also Table E8). However, satisfaction with injectable mAb treatments was high (Fig 4; see also Table E9 in this article’s Online Repository at www.jaci-global.org).

**Fig 5:**
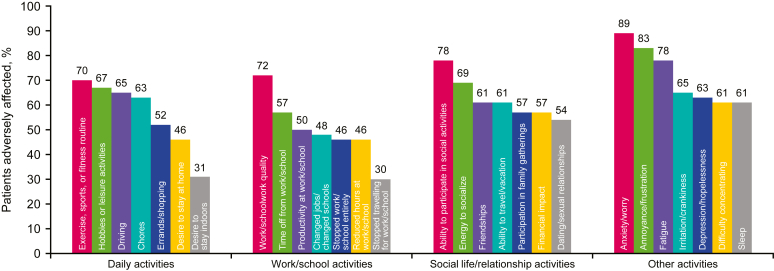
Percentage of respondents reporting an impact of HES on QoL including daily activities, work/school activities, social life/relationship activities, and other activities. Results for items 81 (daily activities), 84 (work/school activities), 87 (social life/relationship activities), and 90 (other activities) of the survey instrument (Table E1). Groups are nonmutually exclusive. Originally presented at the American College of Allergy, Asthma and Immunology 2022 Annual Scientific Meeting.^3^

The most common adverse QoL impacts included ability to exercise, work quality, ability to participate in social activities, and anxiety/worry (Fig 5). Except for errands/shopping, more than 50% of respondents reported issues with daily activities each day or weekly (see Fig E1 in this article’s Online Repository at www.jaci-global.org). At least half of the respondents also experienced daily or weekly issues with work/school productivity (72%) or work/schoolwork quality (51%) or had to stop work/school (50%). Regarding daily or weekly effects on social life and relationship activities, 90% of respondents indicated adverse financial impact, and most respondents reported that dating/sexual relationships (82%), friendships (71%), and energy to socialize (66%) were negatively impacted. Most respondents had fatigue (83%) or their sleep affected (79%) daily or weekly, and most also reported annoyance/frustration (64%), anxiety/worry (63%), or depression/hopelessness (61%) at the same frequency.

Forty-four (83%) respondents “somewhat” or “strongly” agreed that their HES symptoms were burdensome to their family and friends. Forty-two (79%) respondents “somewhat” or “strongly” agreed that they felt different from others or misunderstood because of their HES. Thirty-nine (74%) respondents “somewhat” or “strongly” agreed that HES meant that they were “unable to do certain things for/with [their] family that [they] could do if [they] did not have HES.”

When patients were asked to name one thing that they wanted their doctor to know about their HES, the most common responses were that their HES was real, that they experienced symptoms and pain, that HES was a long-term disease, that it was mentally challenging, and that there was a lack of understanding. A word cloud in which the frequency of use of particular words and phrases is indicated by the size of the text is shown in Fig E2 (in the Online Repository available at www.jaci-global.org); in addition, the patient responses are provided in Table E10 (in the Online Repository available at www.jaci-global.org).

## Discussion

HES is a chronic, multiorgan system disease with a high-impact direct (symptom) and indirect (adverse QoL, medication/treatment, and HCRU) burden requiring a holistic approach to care, yet information is scarce on the lived experience of people with HES. Previously, patient-reported outcome (PRO) tools have been used to identify OCS use in HES, along with adverse effects on QoL, cognitive and behavioral function, emotional well-being, and physical function.^10^ Social media analysis has also identified more personal patient perspectives, such as advice giving/seeking, feeling a lack of control, and negative HCP/health care service experiences.^10^^,^^14^

We believe this is the first study to present in-depth profiling of the patient experience and journey with HES, allowing novel qualitative and quantitative insights into patients’ heterogeneous disease burden. This may enhance HCP recognition, earlier diagnosis, and multidisciplinary management pathways tailored to specific patient needs. However, further research is needed. First, all survey participants were on the APFED group contact list and were English-speaking. The survey was web-based, potentially introducing an age bias or excluding patients unable to access online resources, and its length, associated time to complete the survey, and small honorarium may have limited participation; only 12% of potentially eligible patients completed it. Survey data were confined to the questionnaire responses and were not verified by any other data source, and the study team could not contact respondents to query responses. It should be noted that laboratory data were not included because this was beyond the scope of a survey-centric study, without electronic medical record data available. Finally, longitudinal reports of patient experience with HES, although of ongoing research interest, were out of scope for this study and a potential limitation. These factors could limit the generalizability and reliability of the findings.

The survey identified that many people endure a long, complicated route to obtaining an HES diagnosis, although the survey collected data on patients’ past experiences, which may induce recall bias. However, as supported by social media analysis, in which delayed diagnosis and misdiagnosis of HES was a key concern (82% of posts),^14^ this suggests a lack of HCP education and awareness of the disease. When asked to describe what they would like their doctor to know about their HES, frustrations over the lack of disease recognition were clear, suggesting that people with HES have faced difficulties getting their condition taken seriously by their HCPs. The nonspecificity of the symptoms reported by respondents to this survey also likely contributes to diagnostic challenges.

We identified that people with HES suffer heterogeneous symptoms coupled with a significant disease burden. These findings, supported by previous research using PRO tools to assess prevalence of symptoms among patients with HES,^10^ validate this self-selected cohort,^10^ in which we also examined the burden and frequency of symptoms. Interestingly, the top-reported symptoms (fatigue and general discomfort) do not fully correspond with the most commonly reported eosinophilic comorbidity (asthma), although wheezing and dry cough were among the top 5 symptoms reported. APFED serves patients with all subsets of eosinophil-associated disorders, including a large number with eosinophilic GI disorders based on review of the subscriber database by APFED. In this study, nearly a third of patients reported eosinophilic esophagitis and half reported swallowing difficulty and food impactions, which may suggest an overrepresentation bias of patients with GI disorders compared with other cohorts of patients with HES,^4^^,^^10^^,^^12^ although it has been reported that about 30% of patients with M-HES initially present with GI symptoms.^22^

Comorbid conditions potentially resulting in end-organ damage have a great impact on the QoL of people with HES, particularly eosinophilic asthma and eosinophilic GI diseases. HES had a widespread QoL impact in our survey, supported by the previously published PRO study.^10^ However, this survey provides additional information by including the frequency of adverse QoL impacts, highlighting the pressing need for multidisciplinary HES care including individualized support for psychological needs, fatigue/sleep problems, issues with exercising/physical activity, and support for maintaining employment. The notable impact of HES on working life is highlighted by demographic data showing that less than 40% of respondents were in full-time employment, compared with approximately 70% for the US general population in 2021.^23^ Therefore, it would be of interest to look more closely at the impact of HES on work productivity.

The survey revealed high HCRU, with most patients reporting 1 to 3 hospital admissions for HES over the past 12 months, and multiple visits to primary care physicians, pulmonologists, and allergists/immunologists were typical. To improve symptoms and QoL, more than one-fifth of patients were currently using steroids and half used steroids before HES diagnosis. Despite reported patient satisfaction and improvements in QoL with steroid treatment, this was the most commonly discontinued treatment, possibly reflecting adverse side effects/poor tolerability/steroid toxicity or lack of efficacy (this was not captured directly but may have been reported under the “Other” category).^24^ The burden of OCS use in HES is underrecognized, yet there is a clear dose relationship between OCS use and increased risk of a wide range of complications.^25^ The continued use of OCSs despite their known risks suggests a high disease burden in patients. However, therapies such as anti–IL-5 mAbs can reduce HES flares and OCS use and should provide clinical benefits in this population.^26^^,^^27^

In the United States, mepolizumab is currently the only approved therapy in this drug class for the treatment of HES without an identifiable nonhematologic secondary cause^28^, ^29^, ^30^; other anti–IL-5 and anti–IL-5 receptor biologics are also currently being studied for this population.^31^, ^32^, ^33^, ^34^ Imatinib is approved for Fip1-like 1 platelet-derived growth factor receptor mutated myeloid HES and chronic eosinophilic leukemia.^35^ Most respondents in our survey reported that they received injectable mAb treatment for their HES, for which overall treatment satisfaction was high and linked closely with improved QoL. Unlike steroids, these medicines were not frequently discontinued by HCPs, possibly because of their acceptable tolerance and efficacy.^4^^,^^24^ Because approval of biologics for use in HES is relatively new (the Food and Drug Administration’s approval of mepolizumab for HES occurred about 18 months before this survey was distributed^29^^,^^36^), participants not receiving biologics may not have seen an HCP for their HES since their approval or may have been satisfied with their current treatment. Other therapy options were also used, and more than half of the participants who reported having M-HES were being treated with cyclosporine. This agent is most commonly used to treat L-HES,^37^, ^38^, ^39^ suggesting that patients may not be accurately reporting their disease type. However, there is limited general information around types of HES, which may explain this discrepancy.

Despite treatment satisfaction, this patient-centric approach identified key unmet needs for further study, including symptom burden, treatment burden associated with HES in terms of time taken away from other activities, and the impact of treatment satisfaction/dissatisfaction on symptom burden and QoL. These unmet needs also suggest that the use of targeted therapeutic options could be optimized, and it would be of interest to assess the ability of therapies to address HES symptoms. Notably, in the phase 3 pivotal trial, mepolizumab treatment was associated with significant improvements in fatigue severity versus placebo, measured using the 10-point Brief Fatigue Inventory score.^27^

### Conclusion

This survey study highlights the merit of research partnerships with patient advocacy groups such as APFED, which are uniquely situated to facilitate, support, and coordinate patient-focused research. The results provide a voice for patients with HES and novel insights into the challenges, heterogeneous clinical symptoms, and associated multifactorial burden they face, highlighting the key unmet needs of this population and offering a route toward improved diagnosis and management. In particular, these findings could facilitate individualized treatment toward the most impacted organ systems and may be useful in developing novel PRO measures for HES. Consensus guideline and educational program development incorporating the patient voice could also promote HCP awareness of HES and as such may reduce delays in diagnosis and treatment.

## Disclosure statement

This study was funded by GSK (GSK ID 214158). The sponsor was involved in study design and implementation as well as data collection, analysis, interpretation, writing the study report, and reviewing this article. The sponsor did not place any restrictions on access to data or statements made in the article. All authors had full access to the data on request and had final responsibility for the decision to submit for publication.

Disclosure of potential conflict of interest: J. Silver was formerly employed by GSK and holds financial equities in GSK; and is currently employed by Amgen and holds financial equities in Amgen. A. Kovalszki has received consulting fees from GSK and ALK; has received research funds from Blueprint Medicines and is part of an HES trial with AstraZeneca; is part of the 10.13039/100007270University of Michigan Inhale Collaborative Quality Initiative, sponsored by the Blue Cross Blue Shield of Michigan; and is the Topic Editor at DynaMed for *Eosinophilia: Approach to the Patient*. M. J. Strobel and C. Schmitt are employed by APFED, which received payment from GSK to conduct this study. APFED receives grants and funding to support nonpromotional education, community, and/or research initiatives from Abbott, Ajinomoto Cambrooke, AstraZeneca, Bristol Myers Squibb, Cambrooke, Ellodi, EnteroTrack, EvoEndo, Mead Johnson Nutrition, Nutricia, Regeneron, PhRMA, Revolo, and Sanofi. D. Gratie is employed by AESARA, which received payment from GSK to conduct this study. A. G. Edgecomb was employed by AESARA at the time of the study, which received payment from GSK to conduct this study; and is currently employed by GSK and holds financial equities in GSK. W. Ahmed and A. Deb are employed by GSK and hold financial equities in GSK.
