# Supplementary material for: Burden of hypereosinophilic syndromes in the United States: Patients’ perspective
Source: J Allergy Clin Immunol Glob. 2025 May 28;4(3):100501. doi: 10.1016/j.jacig.2025.100501 (PMC12246599; doi:10.1016/j.jacig.2025.100501)
Supplement: Supplementary Tables [file mmc1.docx]

**Table E1.** HES disease burden survey

| Question 1 | 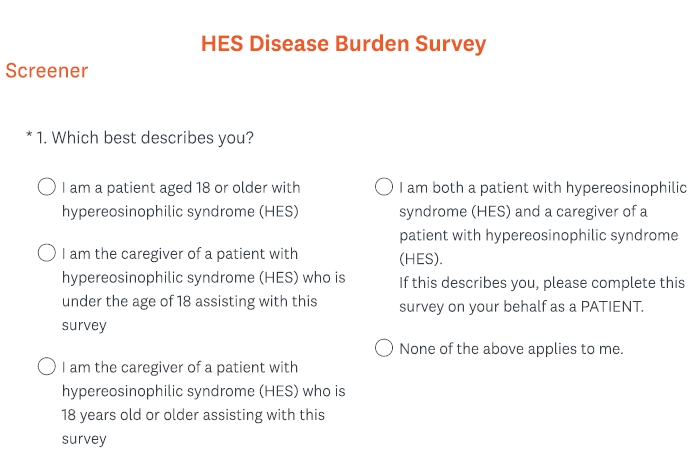 |
| --- | --- |
| Question 2 | 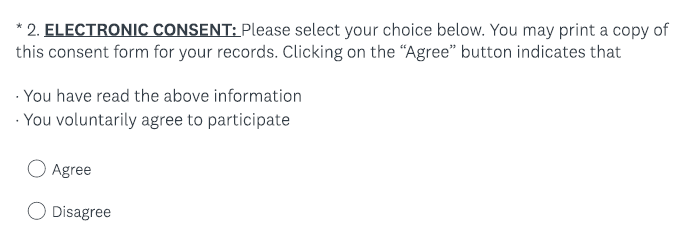 |
| Question 3 | 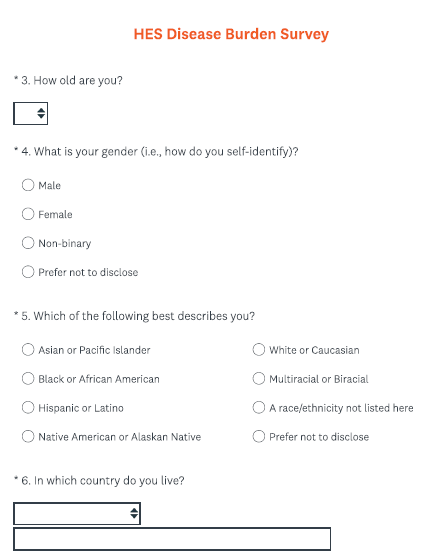 |
| Question 4 | 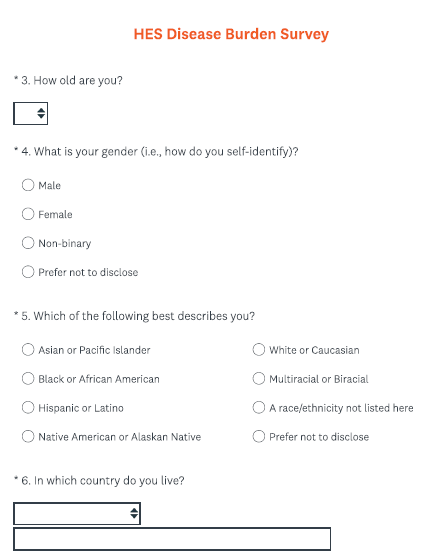 |
| Question 5 | 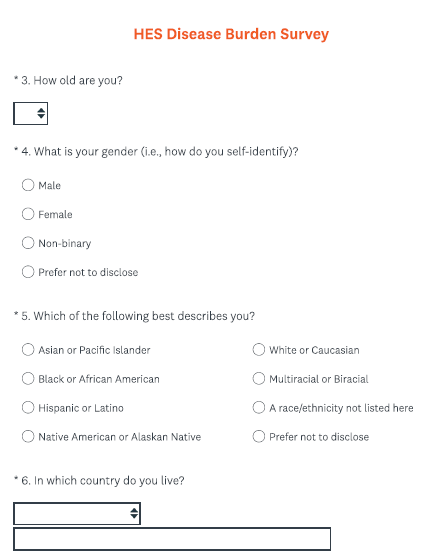 |
| Question 6 | 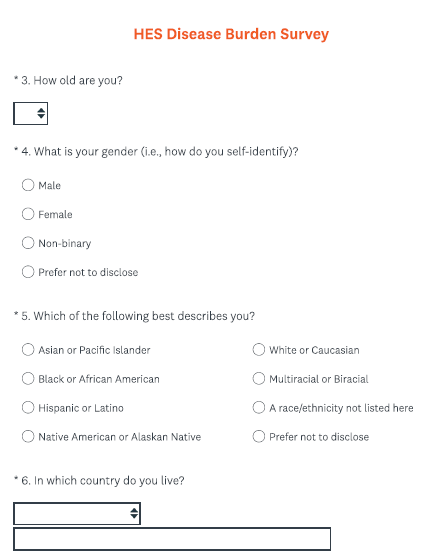 |
| Question 7 | 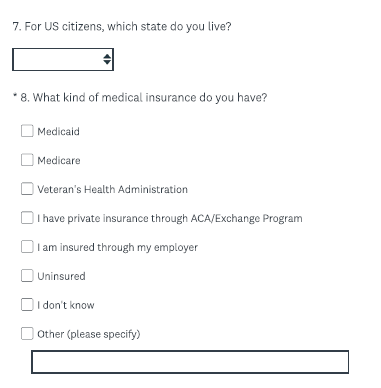 |
| Question 8 | 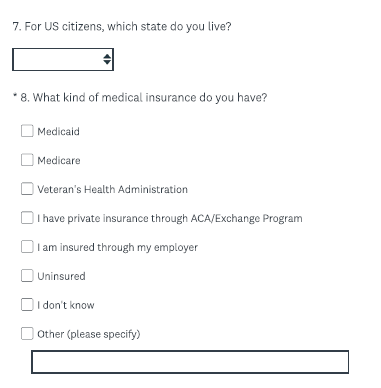 |
| Question 9 | 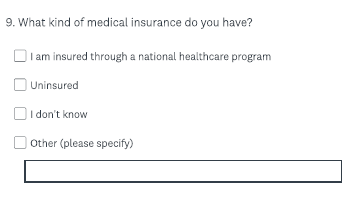 |
| Question 10 | 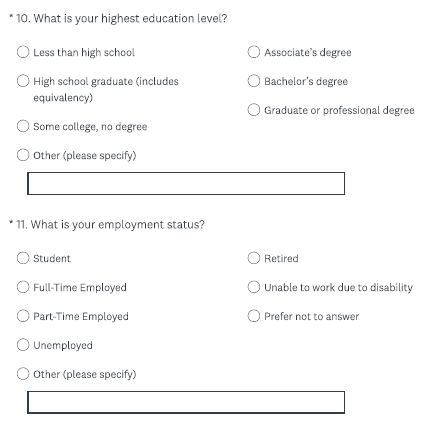 |
| Question 11 | 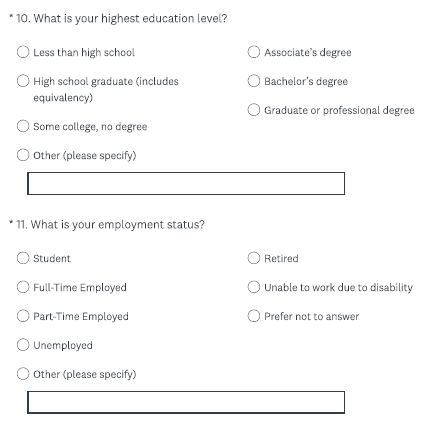 |
| Question 12 | 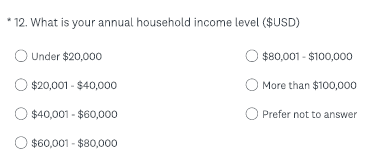 |
| Question 13 | 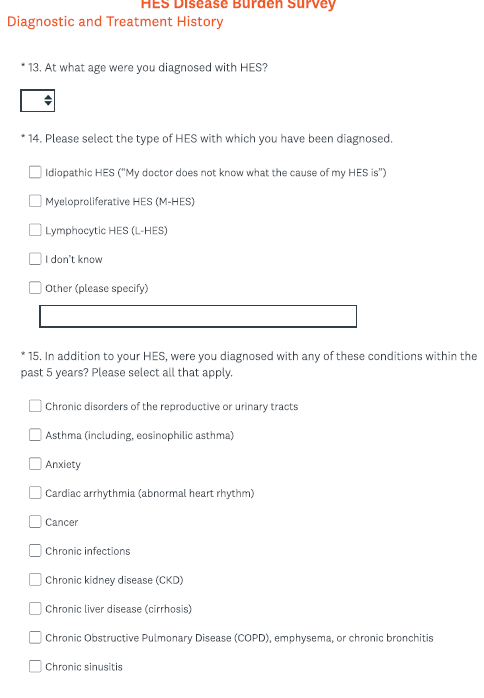 |
| Question 14 | 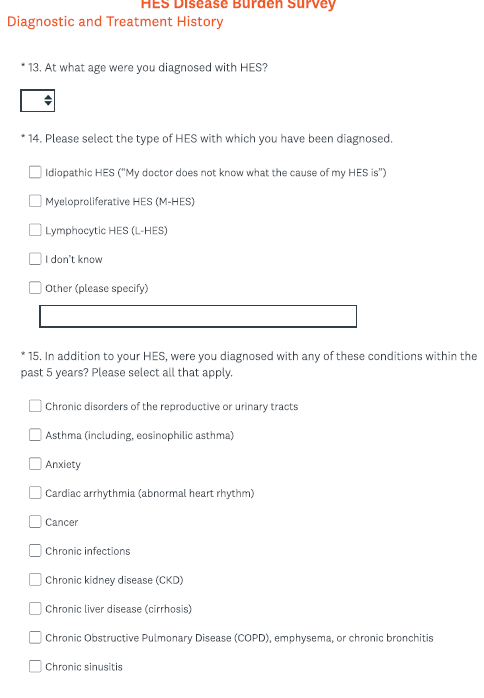 |
| Question 15 | 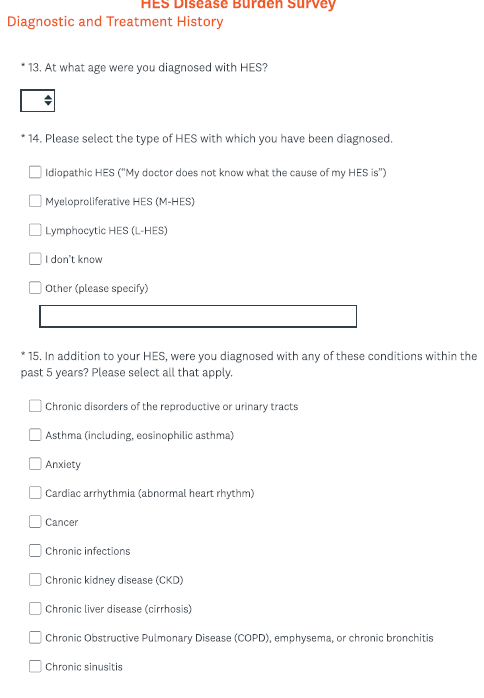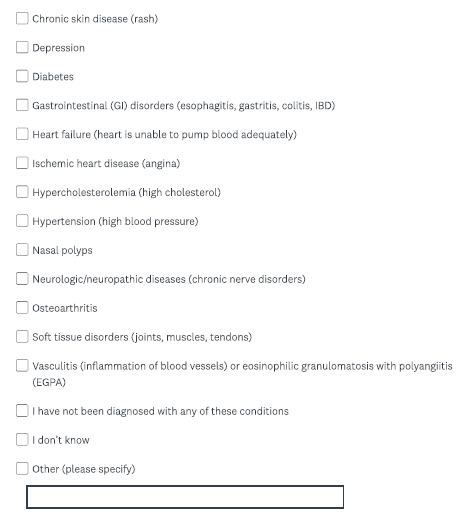 |
| Question 16 | 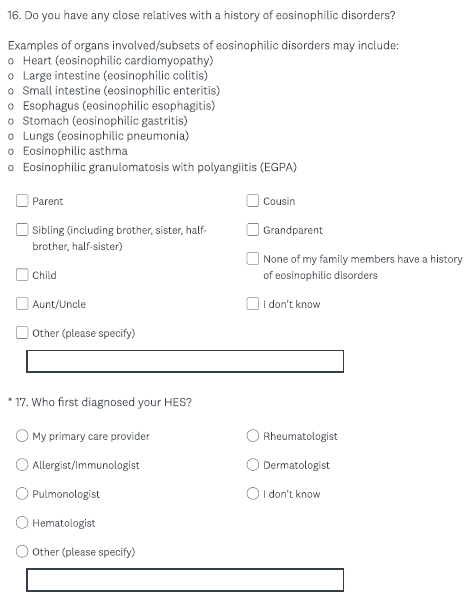 |
| Question 17 | 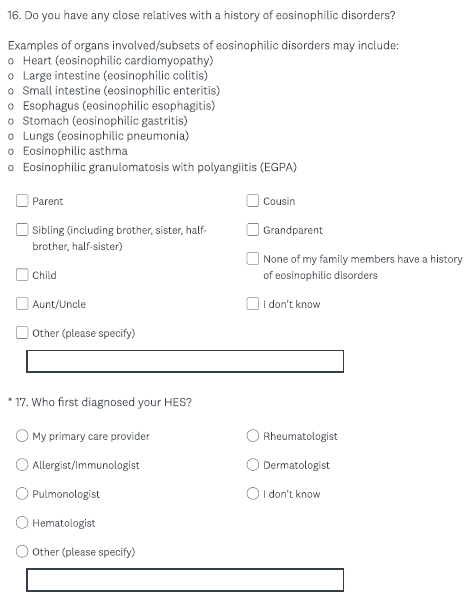 |
| Question 18 | 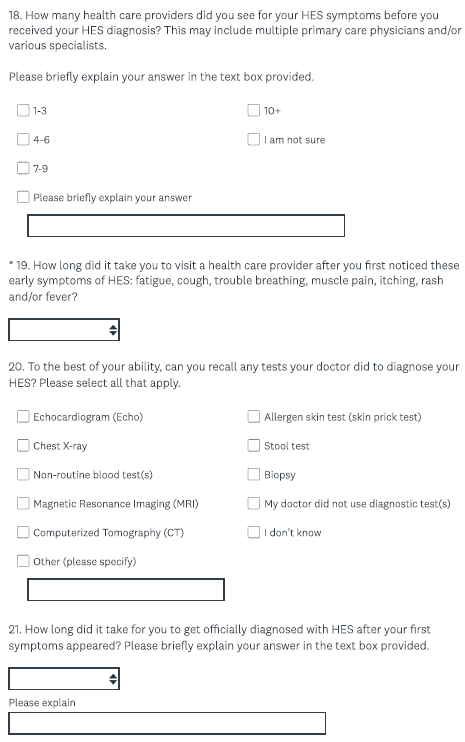 |
| Question 19 | 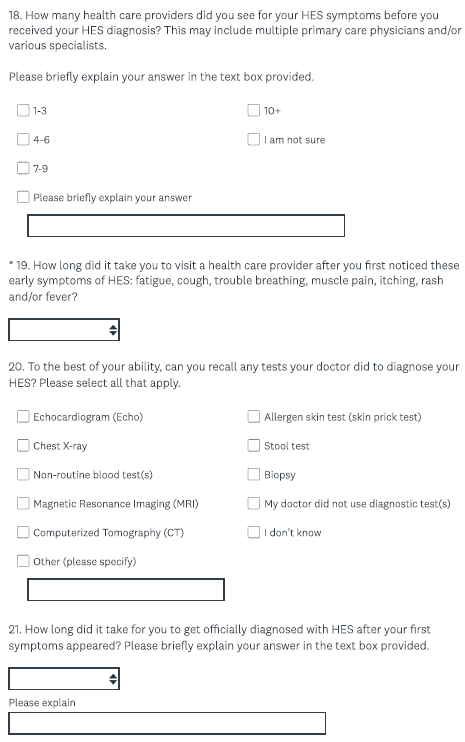 |
| Question 20 | 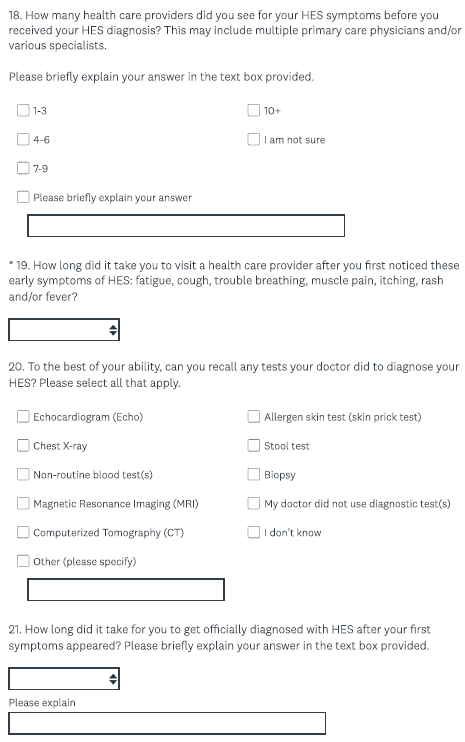 |
| Question 21 | 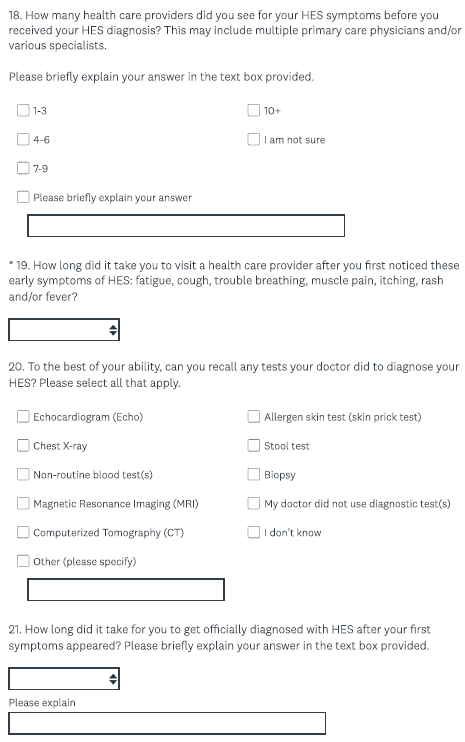 |
| Question 22 | 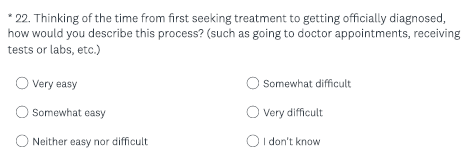 |
| Question 23 | 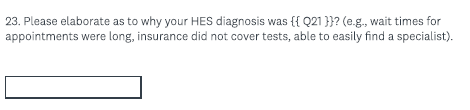 |
| Question 24 | 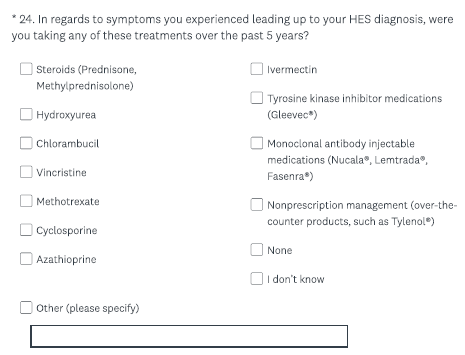 |
| Question 25 | 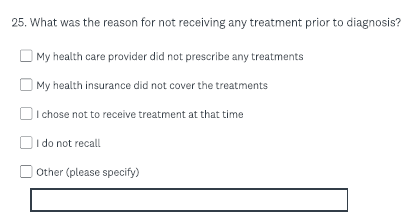 |
| Question 26 | 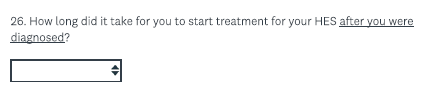 |
| Question 27 | 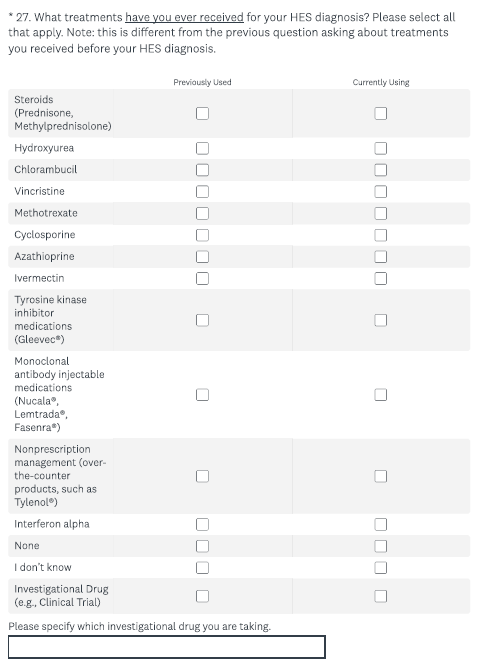 |
| Question 28 | 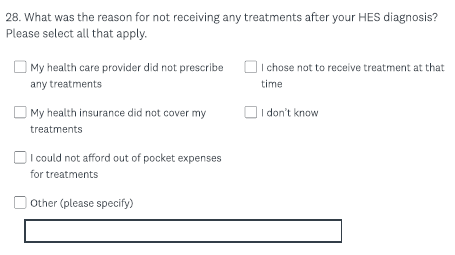 |
| Question 29 | 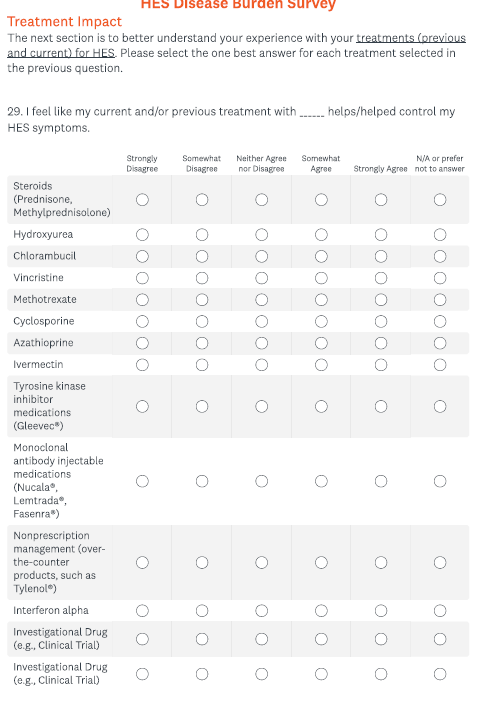 |
| Question 30 | 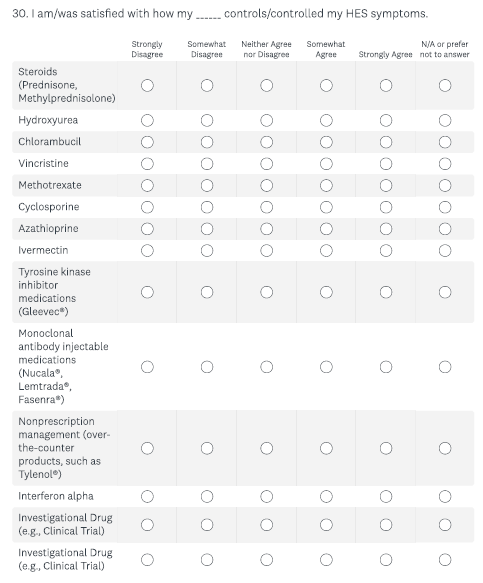 |
| Question 31 | 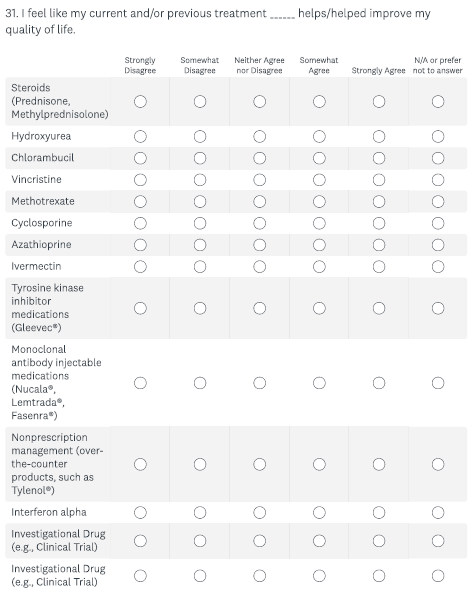 |
| Question 32 | 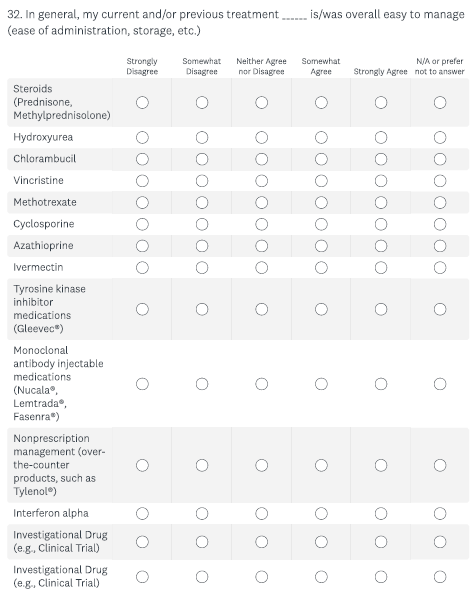 |
| Question 33 | 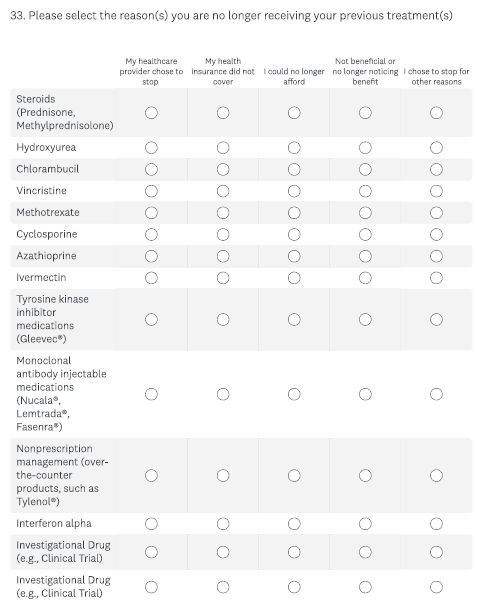 |
| Question 34 | 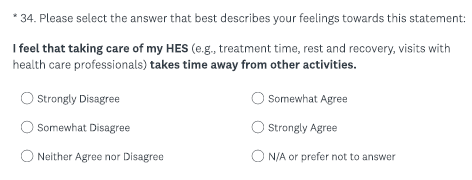 |
| Question 35 | 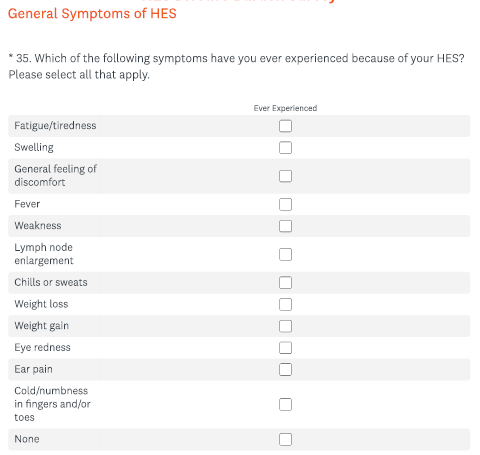 |
| Question 36 | 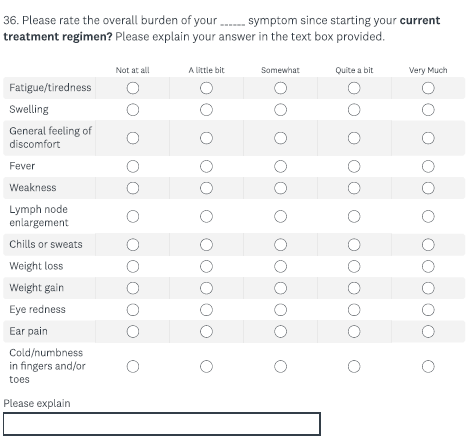 |
| Question 37 | 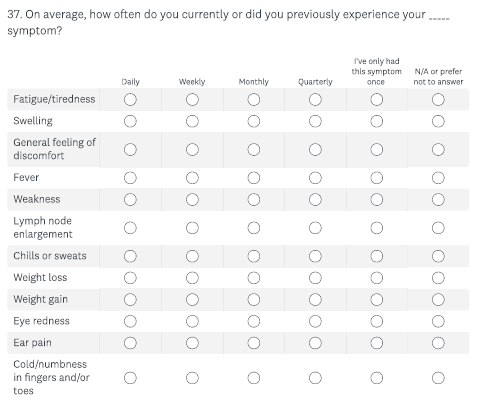 |
| Question 38 | 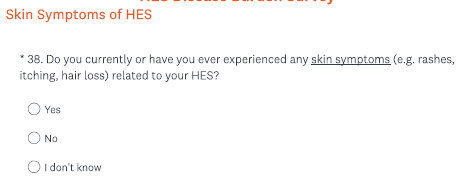 |
| Question 39 | 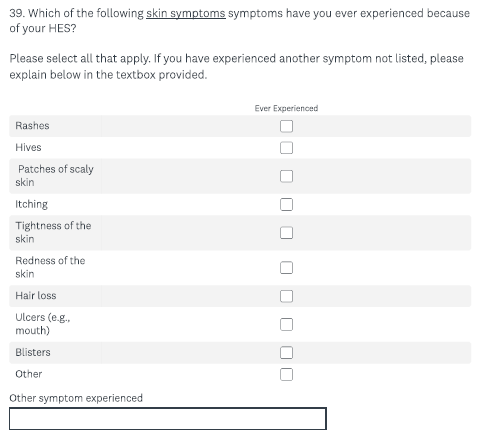 |
| Question 40 | 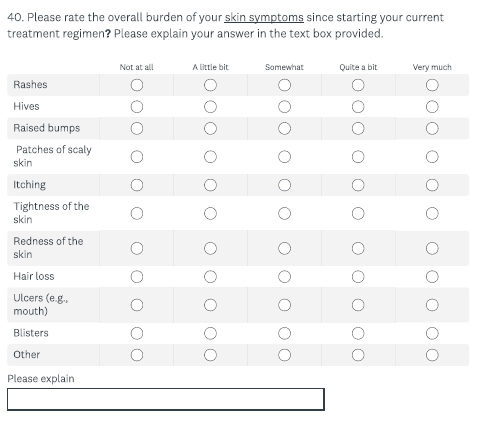 |
| Question 41 | 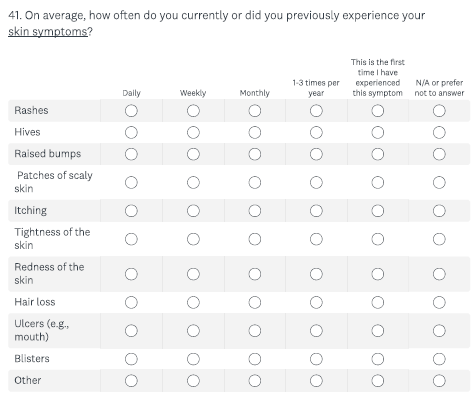 |
| Question 42 | 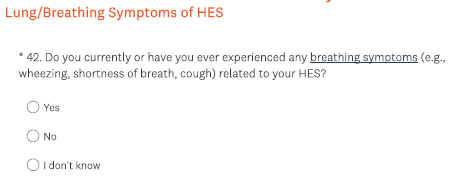 |
| Question 43 | 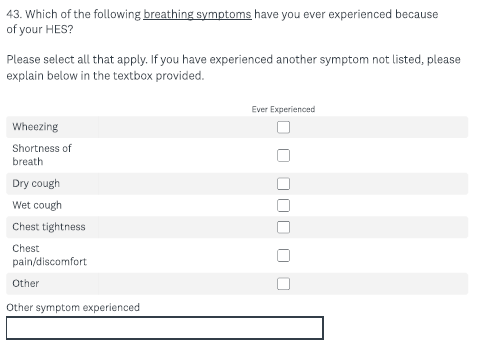 |
| Question 44 | 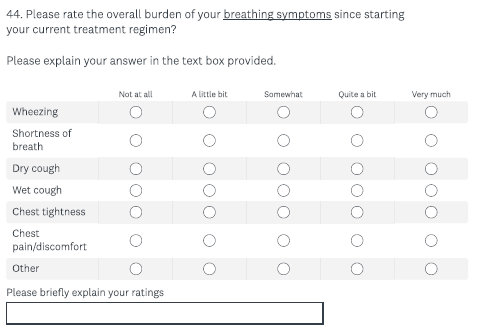 |
| Question 45 | 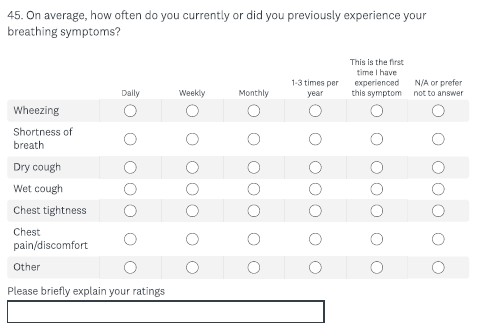 |
| Question 46 | 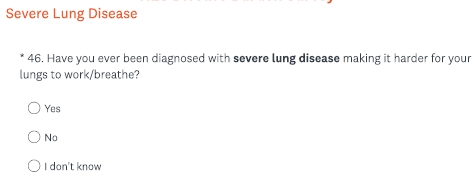 |
| Question 47 | 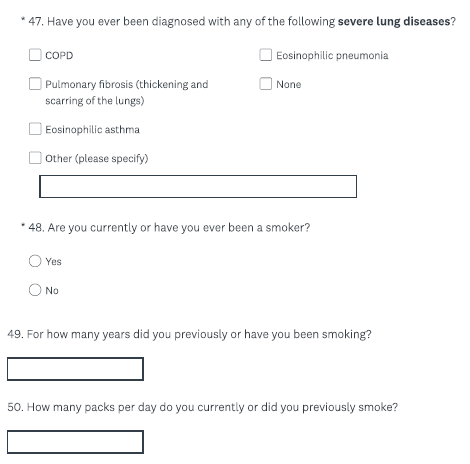 |
| Question 48 | 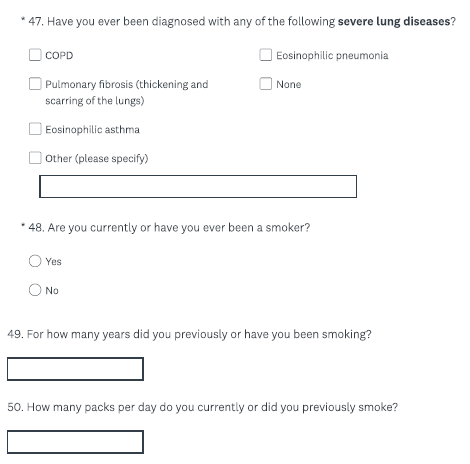 |
| Question 49 | 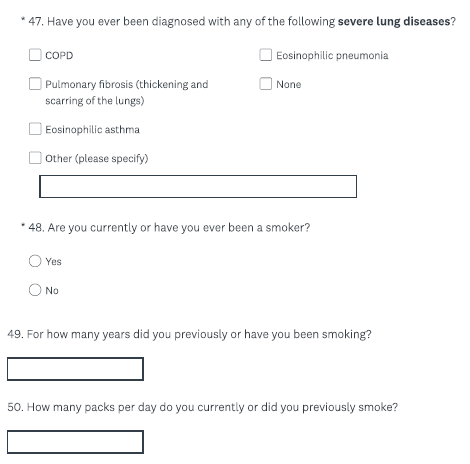 |
| Question 50 | 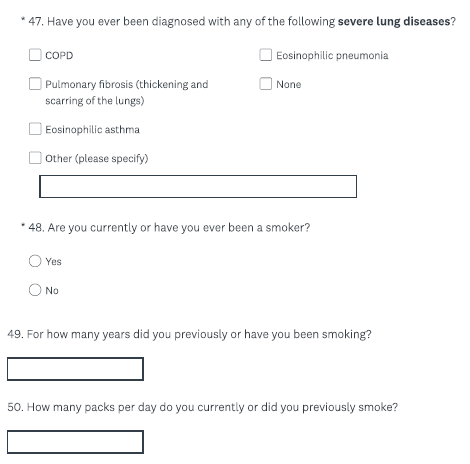 |
| Question 51 | 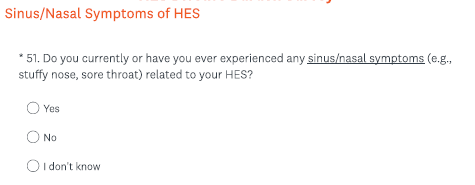 |
| Question 52 | 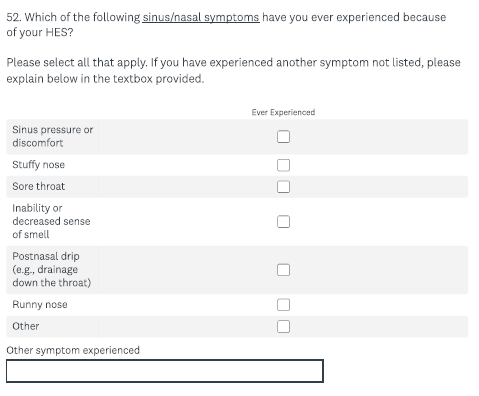 |
| Question 53 | 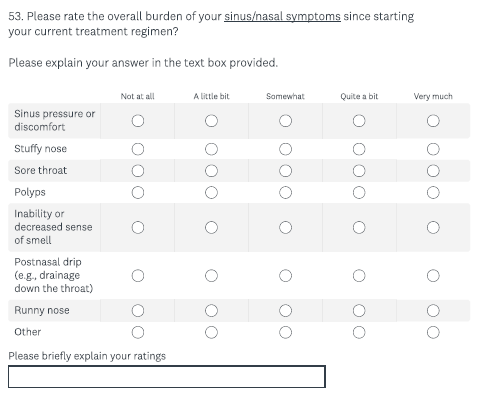 |
| Question 54 | 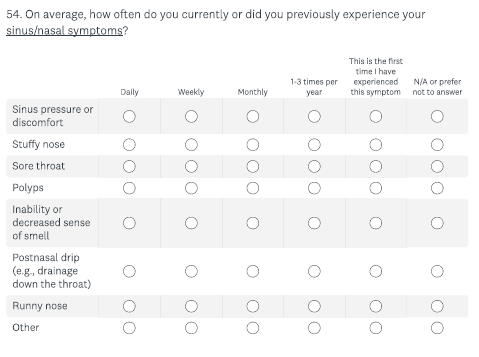 |
| Question 55 | 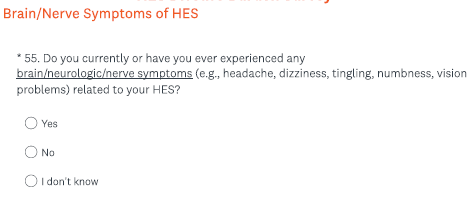 |
| Question 56 | 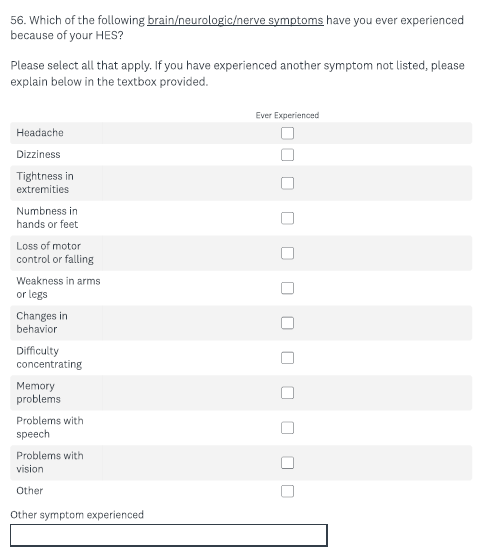 |
| Question 57 | 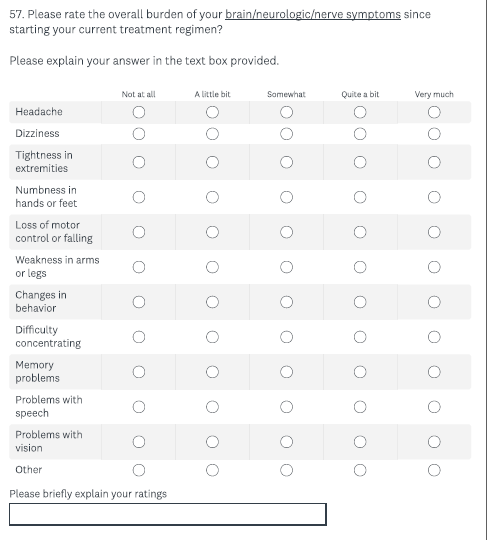 |
| Question 58 | 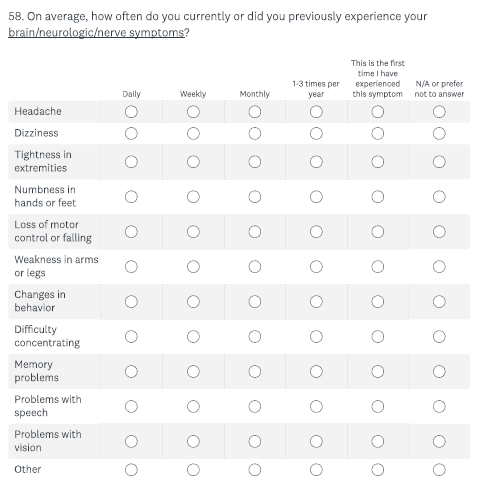 |
| Question 59 | 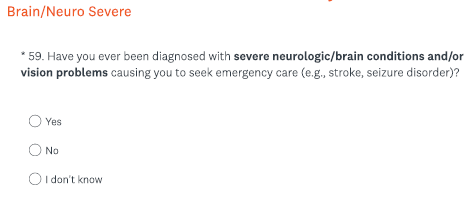 |
| Question 60 | 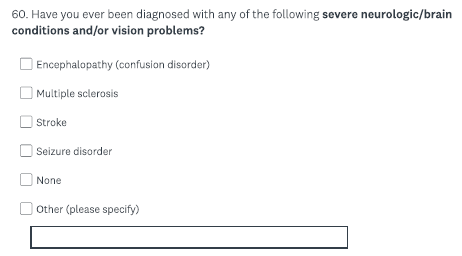 |
| Question 61 | 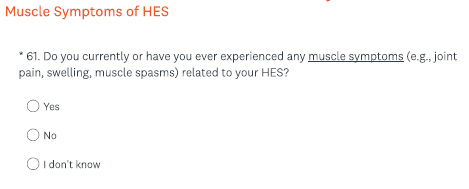 |
| Question 62 | 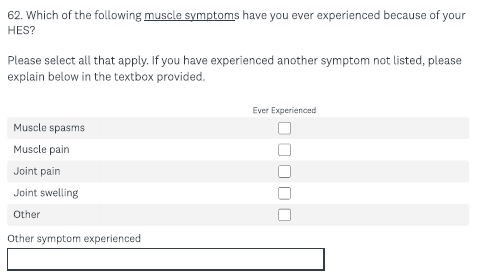 |
| Question 63 | 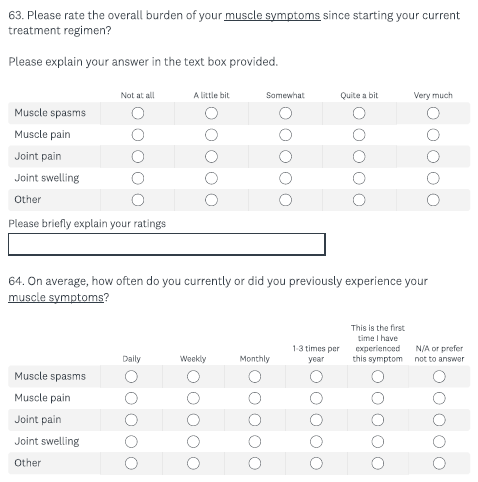 |
| Question 64 | 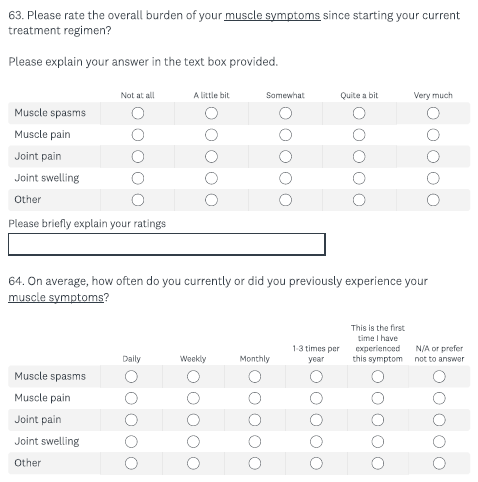 |
| Question 65 | 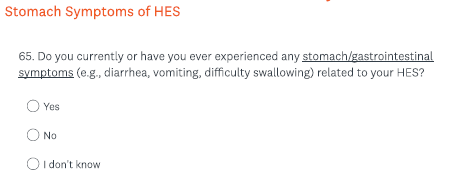 |
| Question 66 | 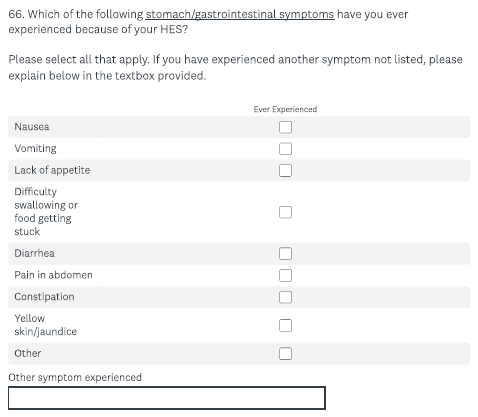 |
| Question 67 | 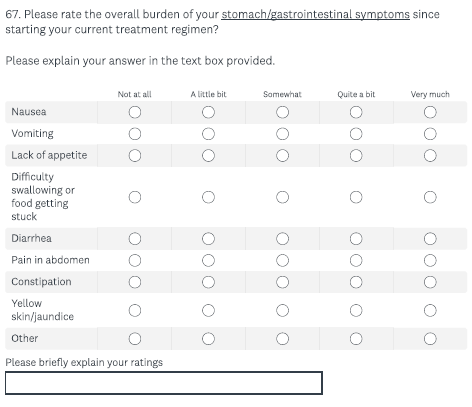 |
| Question 68 | 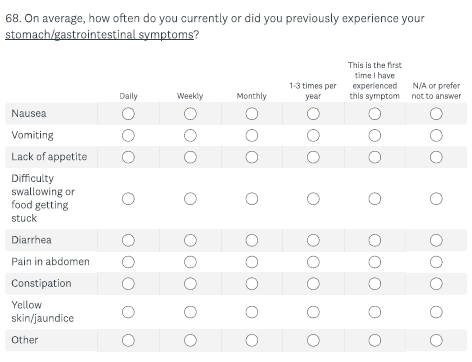 |
| Question 69 | 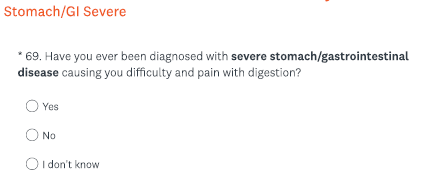 |
| Question70 | 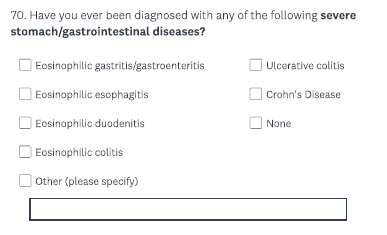 |
| Question 71 | 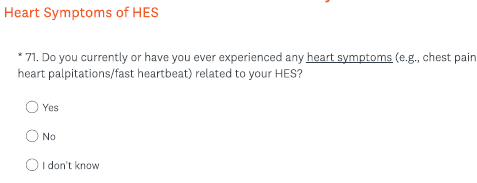 |
| Question 72 | 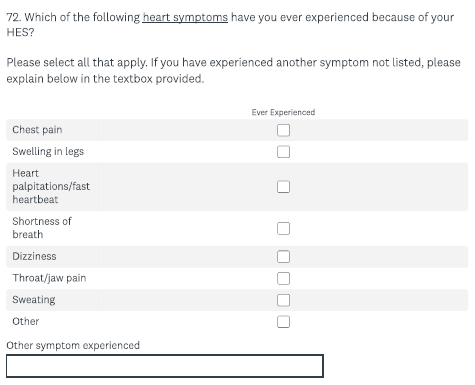 |
| Question 73 | 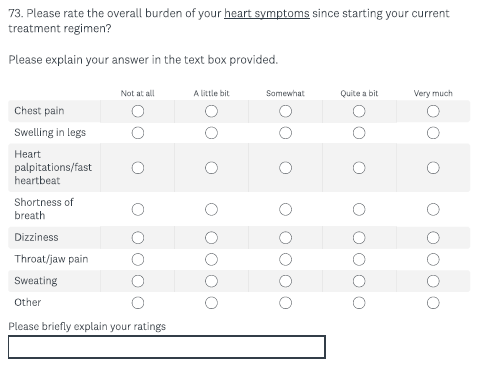 |
| Question 74 | 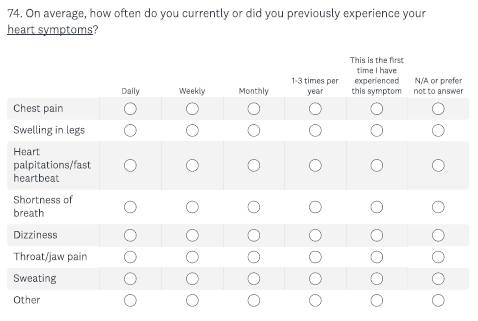 |
| Question 75 | 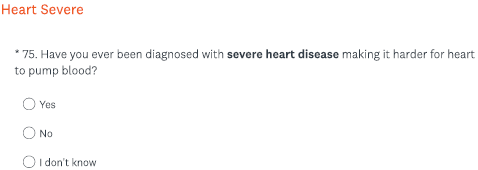 |
| Question 76 | 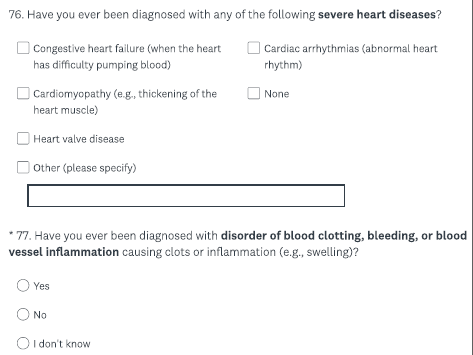 |
| Question 77 | 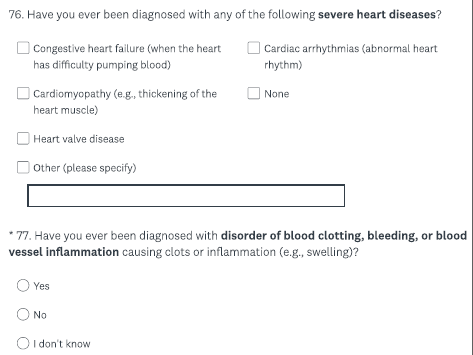 |
| Question 78 | 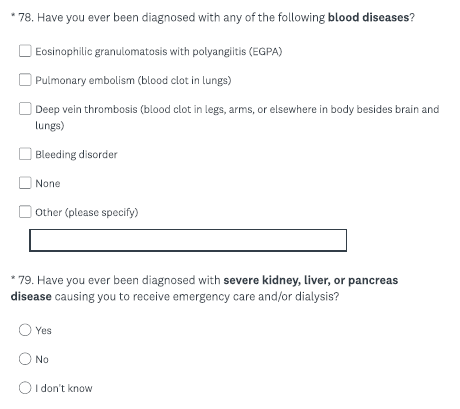 |
| Question 79 | 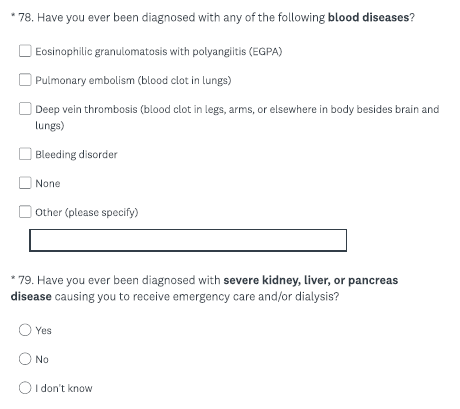 |
| Question 80 | 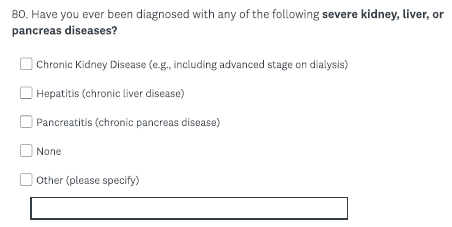 |
| Question 81 | 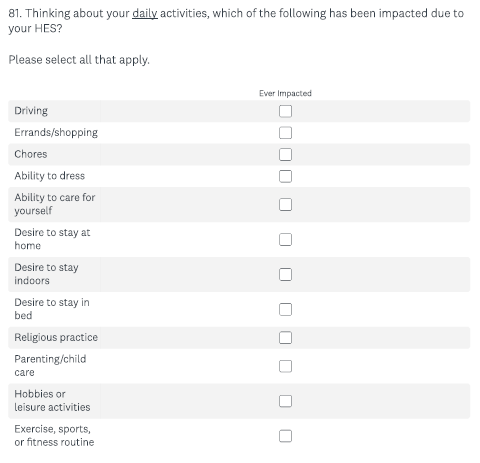 |
| Question 82 | 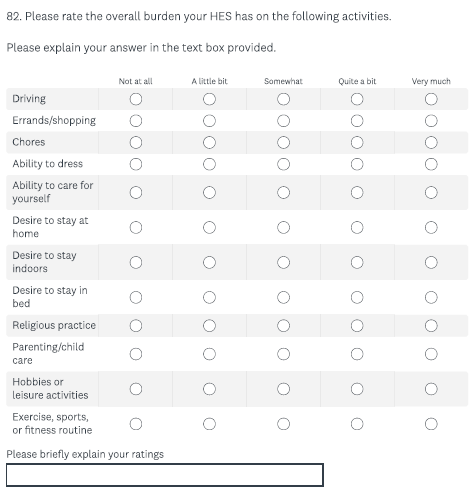 |
| Question 83 | 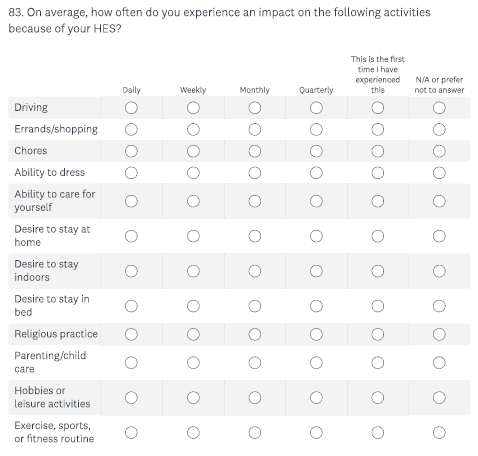 |
| Question 84 | 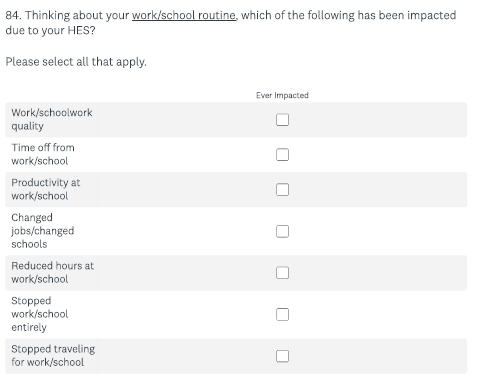 |
| Question 85 | 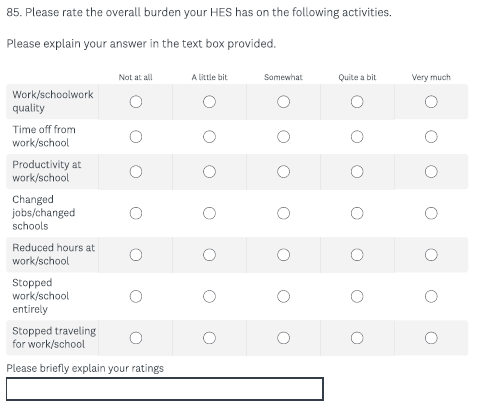 |
| Question 86 | 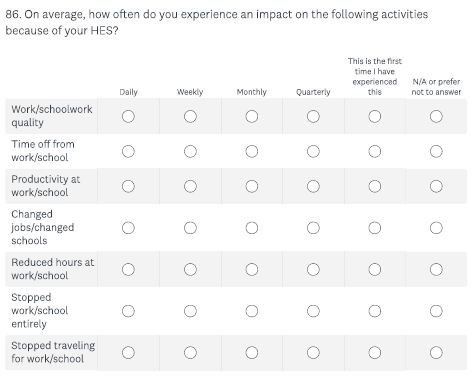 |
| Question 87 | 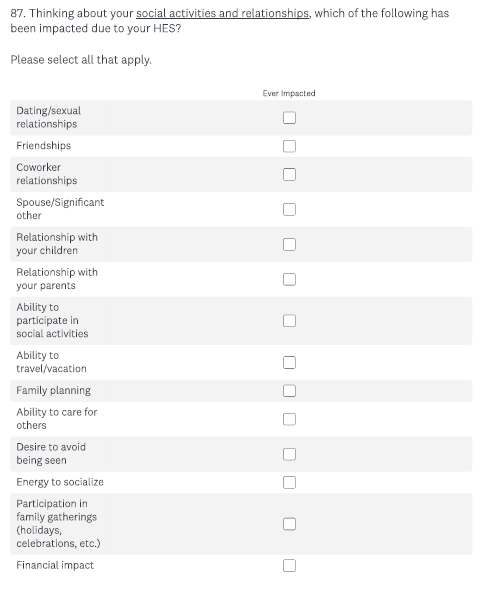 |
| Question 88 | 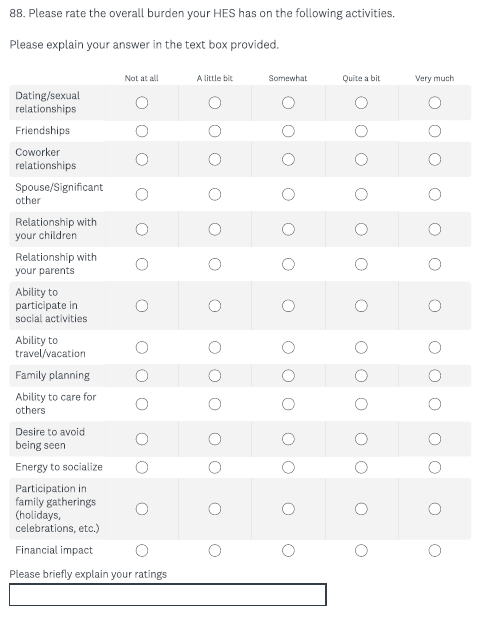 |
| Question 89 | 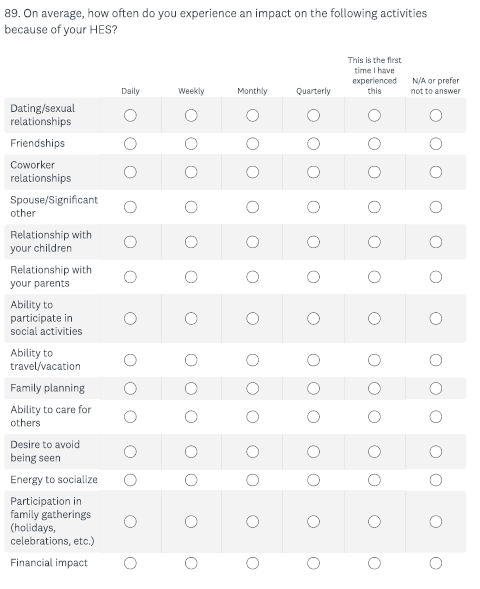 |
| Question 90 | 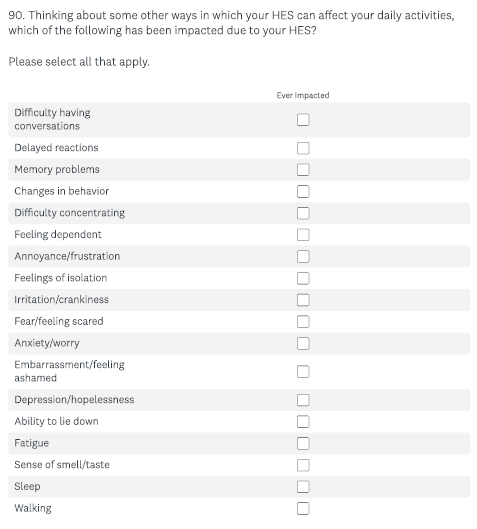 |
| Question 91 | 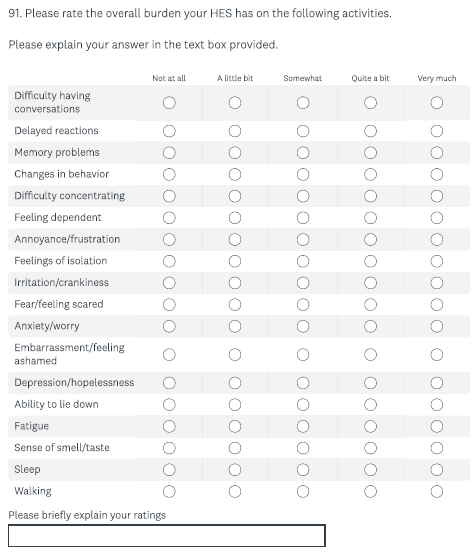 |
| Question 92 | 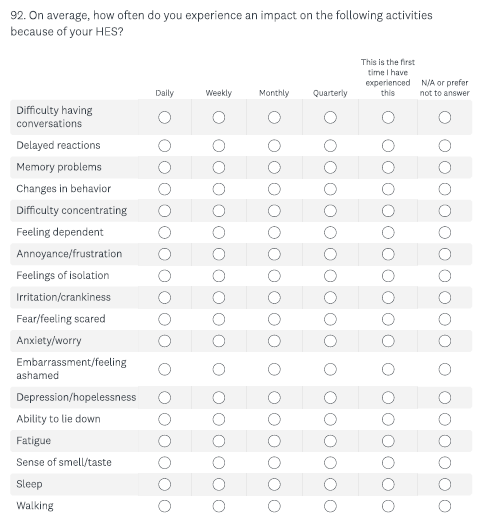 |
| Question 93 | 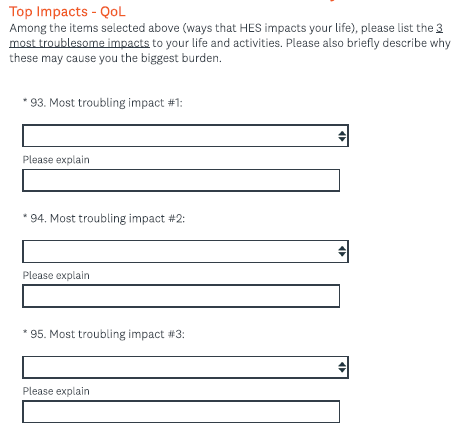 |
| Question 94 | 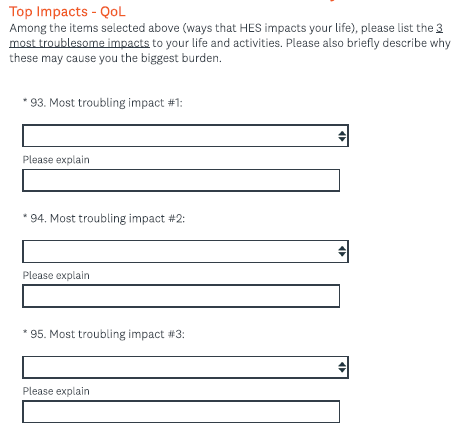 |
| Question 95 | 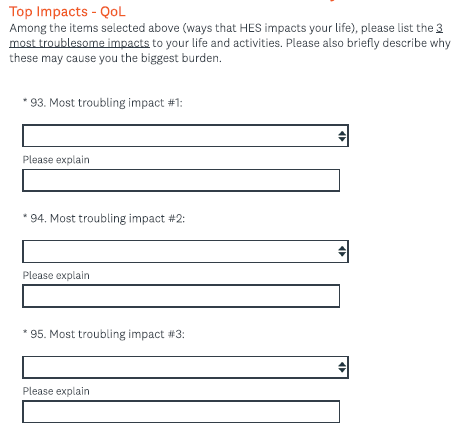 |
| Question 96 | 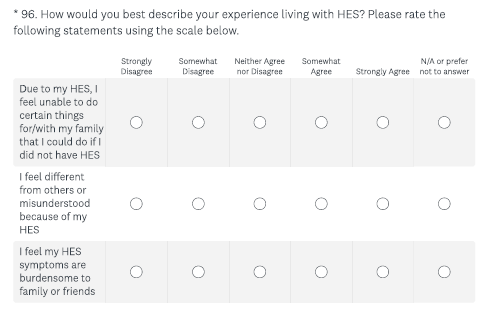 |
| Question 97 | 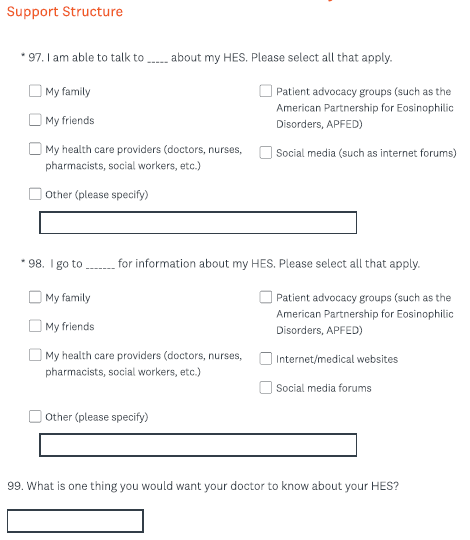 |
| Question 98 | 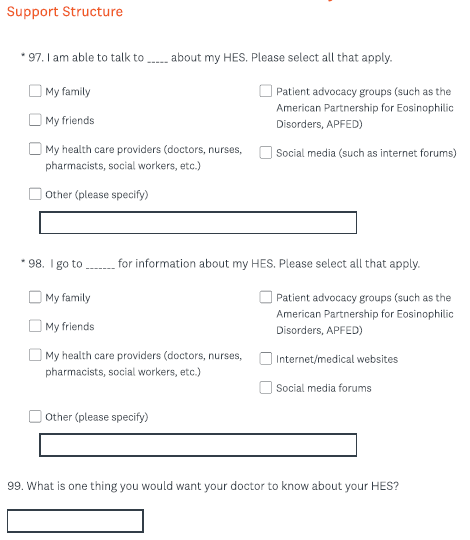 |
| Question 99 | 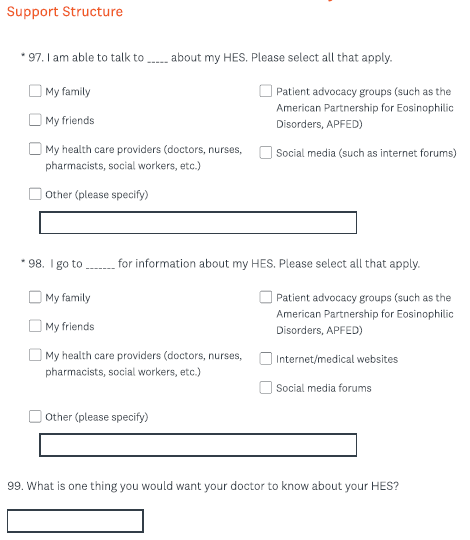 |
| Question 100 |  |
| Question 101 |  |
| Question 102 |  |
| Question 103 |  |
| Question 104 |  |

### **Table E2.** Patient demographics

| **Demographics (US cohort)** | | |
| --- | --- | --- |
| **Total number of respondents, N** | 54 | |
| Patients, n (%) | 53 (98) | |
| Caregiver on behalf, n (%) | 1 (2) | |
| **Age, years** | | |
| Age, mean ± SD | 43.6 ± 14.0 | |
| Age, median (Min, Max) | 42 (14, 82) | |
| **Age group, years** | **n** | **%** |
| <18 | 1 | 2 |
| 18─44 | 32 | 59 |
| 45─64 | 14 | 26 |
| 65+ | 7 | 13 |
| **Gender** | **n** | **%** |
| Female/Male | 23/31 | 43/57 |
| **Race/ethnicity** | **n** | **%** |
| Asian or Pacific Islander | 1 | 2 |
| Black or African American | 4 | 7 |
| Hispanic or Latino | 4 | 7 |
| Native American or Alaskan Native | 2 | 4 |
| White or Caucasian | 43 | 80 |
| **Medical insurance coverage*** | **n** | **%** |
| Medicare | 22 | 41 |
| Medicaid | 15 | 28 |
| Commercial† | 22 | 41 |
| Uninsured | 1 | 2 |
| Other^‡^ | 1 | 2 |
| **Highest education level** | **n** | **%** |
| Less than high school | 1 | 2 |
| High school graduate (includes equivalency) | 5 | 9 |
| Associate degree | 20 | 37 |
| Bachelor's degree | 8 | 15 |
| Graduate or professional degree | 7 | 13 |
| Some college, no degree | 13 | 24 |
| **Employment status** | **n** | **%** |
| Full-time employed | 21 | 39 |
| Part-time employed | 9 | 17 |
| Unemployed | 7 | 13 |
| Unable to work due to disability | 8 | 15 |
| Other^§^ | 9 | 17 |
| **Annual household income** | **n** | **%** |
| Less than $20,000 | 4 | 7 |
| $20,000–$60,000 | 15 | 28 |
| $60,000–$100,000 | 18 | 33 |
| More than $100,000 | 10 | 19 |
| No response | 7 | 13 |

Includes responses to items 3–12 of the survey instrument (**Table E1**). *Non‑mutually exclusive groups. †Includes employer and private insurance coverage. ^‡^Includes national healthcare insurance coverage (such as Veterans Health Administration or country-specific national healthcare coverage). ^§^Includes retired status and student.
Abbreviation: SD, standard deviation.

### **Table E3.** HES diagnosis history

| **Diagnosis history (N=54)** | | |
| --- | --- | --- |
| Age at HES diagnosis, mean ± SD, years | 37.11 (15) | |
| Age at HES diagnosis, median (Min, Max) | 36 (5, 80) | |
| **Age group at HES diagnosis, years** | **n** | **%** |
| <18 | 5 | 9 |
| 18─44 | 36 | 67 |
| 45─64 | 11 | 20 |
| 65+ | 2 | 4 |
| **HES diagnosis specialty** | **n** | **%** |
| Primary care | 12 | 22 |
| Allergist/immunologist | 14 | 26 |
| Pulmonologist | 10 | 19 |
| Hematologist | 14 | 26 |
| Rheumatologist | 2 | 4 |
| Other* | 2 | 4 |
| **Number of HCPs visited  (leading to HES diagnosis)** | **n** | **%** |
| 1─3 | 16 | 30 |
| 4─6 | 17 | 31 |
| 7─9 | 12 | 22 |
| ≥10 | 9 | 17 |
| **Time to first visit with HCP  (leading to HES diagnosis)** | **n** | **%** |
| <3 months | 33 | 61 |
| 3–6 months | 8 | 15 |
| 6 months–1 year | 3 | 6 |
| 1–3 years | 6 | 11 |
| 3–5 years | 1 | 2 |
| 5–7 years | 0 | 0 |
| 7–10 years | 1 | 2 |
| >10 years | 2 | 4 |
| **Time from first symptom to HES diagnosis** | **n** | **%** |
| <3 months | 14 | 26 |
| 3–6 months | 5 | 9 |
| 6 months–1 year | 11 | 20 |
| 1–3 years | 12 | 22 |
| 3–5 years | 8 | 15 |
| 5–7 years | 1 | 2 |
| 7–10 years | 1 | 2 |
| >10 years | 2 | 4 |
| **Tests leading to HES diagnosis**^†^ | **n** | **%** |
| Echocardiogram | 18 | 33 |
| Chest x-ray | 19 | 35 |
| Non-routine blood test(s) | 35 | 65 |
| MRI | 32 | 59 |
| CT | 29 | 54 |
| Allergen skin test | 25 | 46 |
| Stool test | 15 | 28 |
| Biopsy | 17 | 31 |
| None | 1 | 2 |
| Other^‡^ | 4 | 7 |
| **Overall satisfaction with the process of HES diagnosis** | **n** | **%** |
| Very easy | 0 | 0 |
| Somewhat easy | 5 | 9 |
| Neither easy nor difficult | 16 | 30 |
| Somewhat difficult | 12 | 22 |
| Very difficult | 20 | 37 |
| Unsure | 1 | 2 |

Includes responses to items 13, and 17–22 of the survey instrument (**Table E1**). *Includes cardiologist, gastroenterologist, neurologist. ^†^Non-mutually exclusive groups. ^‡^Includes spinal tap, endoscopy, colonoscopy, psychological tests, and pulmonary function tests.

Abbreviations: CT, computerized tomography; HCP, healthcare provider; HES, hypereosinophilic syndromes; MRI, magnetic resonance imaging; SD, standard deviation.

### **Table E4.** Clinical characteristics

| **Clinical characteristics** | **All respondents (N=54)** | | |
| --- | --- | --- | --- |
| **Patient-reported HES subtype** | **n** | | **%** |
| Idiopathic | 32 | | 59 |
| M-HES | 15 | | 28 |
| L-HES | 3 | | 6 |
| Other/unsure | 5 | | 9 |
| **Family history of eosinophilic disorders*** | | | |
| Parent | 10 | | 19 |
| Child | 1 | | 2 |
| Other^†^ | 7 | | 13 |
| None | 28 | | 52 |
| Unsure | 11 | | 20 |
| **Comorbidities (diagnosed in past 5 years)*** | | | |
| Asthma^‡^ | 29 | | 54 |
| Anxiety | 14 | | 26 |
| Chronic skin disease | 13 | | 24 |
| Gastrointestinal disorders^§^ | 12 | | 22 |
| None | 11 | | 20 |
| COPD, emphysema, or chronic bronchitis | 8 | | 15 |
| Chronic sinusitis | 7 | | 13 |
| Vasculitis or EGPA | 7 | | 13 |
| Other^¶^ | 7 | | 13 |
| Soft tissue disorders | 6 | | 11 |
| Neurologic/neuropathic diseases | 6 | | 11 |
| Nasal polyps | 5 | | 9 |
| Hypertension | 5 | | 9 |
| Depression | 5 | | 9 |
| Chronic infections | 5 | | 9. |
| Osteoarthritis | 4 | | 7 |
| Hypercholesterolemia | 4 | | 7 |
| Diabetes | 4 | | 7 |
| Chronic liver disease (including NASH, NAFLD) | 4 | | 7 |
| Chronic kidney disease | 4 | | 7 |
| Cancer | 4 | | 7 |
| Heart failure | 2 | | 4 |
| Chronic disorders of the reproductive or urinary tracts | 2 | | 4 |
| Cardiac arrhythmias | 2 | | 4 |
| Unsure | 1 | | 2 |
| Ischemic heart disease | 1 | | 2 |
| **Presence of eosinophilic comorbidities/co-diagnoses*** |  | | |
| **Respiratory** | | | |
| Eosinophilic asthma | 20 | 37 | |
| Current or former smoker** | 6 | 11 | |
| Eosinophilic pneumonia | 4 | 7 | |
| COPD | 3 | 6 | |
| **Gastrointestinal** | | | |
| Eosinophilic esophagitis | 17 | 31 | |
| Eosinophilic gastritis/gastroenteritis | 11 | 20 | |
| Eosinophilic colitis | 2 | 4 | |
| Eosinophilic duodenitis | 1 | 2 | |
| **Vascular** | | | |
| EGPA | 7 | 13 | |

Includes responses to items 14, 15, 16, 47, 70, and 78 of the survey instrument (**Table E1**).
*Non-mutually exclusive groups. †Includes sibling (brother, sister, half-brother, half-sister), aunt/uncle, cousin, and grandmother/grandfather. ^‡^Respondent included “eosinophilic asthma” as HES diagnosis type. ^§^Such as esophagitis, gastritis, colitis, IBD. ^¶^Includes Fukuyama congenital muscular dystrophy-related disorders, mast cell activation syndrome, hyperthyroidism, osteopenia/osteoporosis, pleural effusion, Sjogren’s syndrome, swollen lymph nodes, abnormal skin growth and dry eyes. **Of those (n=6) who are current or former smokers, duration of smoking reported ranged from 2 to 20 years with 1–2 packs per day reported.
Abbreviations: COPD, chronic obstructive pulmonary disease; EGPA, eosinophilic granulomatosis with polyangiitis; HES, hypereosinophilic syndromes; IBD, inflammatory bowel disease; L-HES, lymphocytic HES; M-HES, myeloproliferative HES; NAFLD, non-alcoholic fatty liver disease; NASH, non-alcoholic steatohepatitis.

### **Table E5.** HES comorbidities and complications by organ class

| **HES comorbidities and complications*** | **Patients with HES (N=54)** |
| --- | --- |
| **Lungs** |  |
| **Comorbidities, n (%)** |  |
| Asthma | 29 (54) |
| Eosinophilic asthma | 20 (37) |
| Current or former smoker^†^ | 6 (11) |
| Eosinophilic pneumonia | 4 (7) |
| COPD | 3 (6) |
| Pulmonary fibrosis | 1 (2) |
| Other^‡^ | 1 (2) |
| None | 2 (4) |
| **Brain** |  |
| **Comorbidities, n (%)** |  |
| Encephalopathy | 3 (6) |
| Multiple sclerosis | 1 (2) |
| Parkinson’s disease^§^ | 1 (2) |
| None | 1 (2) |
| **Complications, n (%)** |  |
| Stroke or TIA | 2 (4) |
| Seizure | 1 (2) |
| **Gastrointestinal tract** |  |
| **Comorbidities, n (%)** |  |
| Eosinophilic esophagitis | 17 (31) |
| Eosinophilic gastritis/gastroenteritis | 11 (20) |
| Eosinophilic colitis | 2 (4) |
| Ulcerative colitis | 2 (4) |
| Eosinophilic duodenitis | 1 (2) |
| Crohn's disease | 1 (2) |
| Other^║^ | 1 (2) |
| None | 1 (2) |
| **Heart and blood** |  |
| **Comorbidities, n (%)** |  |
| Congestive heart failure | 2 (4) |
| Cardiomyopathy | 2 (4) |
| Cardiac arrhythmias | 2 (4) |
| Heart valve disease | 1 (2) |
| None | 1 (2) |
| **Complications, n (%)** |  |
| Deep vein thrombosis | 7 (13) |
| Bleeding disorder (any kind) | 2 (4) |
| Pulmonary embolism | 1 (2) |
| None | 4 (7) |
| Other** | 3 (6) |
| **Other diagnoses, n (%)** |  |
| EGPA | 7 (13) |
| **Kidney, liver, pancreas** |  |
| **Comorbidities, n (%)** |  |
| Chronic kidney disease | 3 (6) |
| Pancreatitis | 3 (6) |
| Hepatitis (any kind) | 2 (4) |
| None | 1 (2) |
| Other^††^ | 1 (2) |
| **Skin**^‡‡^ |  |
| **Complications, n (%)** |  |
| Rash | 44 (81) |
| Hives | 21 (39) |
| Patches of scaly skin | 20 (37) |

Responses to items 47, 60, 70, 76, 78, and 80 of the survey instrument (**Table E1**).

*Non-mutually exclusive groups. ^†^Of those (n=6) who are current or former smokers, duration of smoking reported ranged from 2 to 20 years with 1─2 packs per day reported. ^‡^Includes pneumonia, pleural effusion, and eosinophilic bronchitis. §Submitted as free-text in the “Other (please specify)” option in item 60 of the survey. ^║^Includes GERD and diverticulitis. **Includes thrombocytopenia, Henoch–Schönlein purpura, and iron deficiency anemia. ^††^Includes non-alcoholic steatohepatitis. ^‡‡^Skin comorbidity data were not collected per se but these complications are clear indicators of dermatologic comorbidities such as dermatitis, eczema etc.

Abbreviations: COPD, chronic obstructive pulmonary disease; EGPA, eosinophilic granulomatosis with polyangiitis; GERD, gastro-esophageal reflux disease; HES, hypereosinophilic syndromes; TIA; transient ischemic attack.

### **Table E6.** Hypereosinophilic symptom frequency

| **HES symptom frequency*  (US respondents, N=54)** |  | **Daily** | **Weekly** | **Monthly** | **Quarterly** | **First time experienced** | **N/A or prefer  not to answer** | **Total N** |
| --- | --- | --- | --- | --- | --- | --- | --- | --- |
| ***General symptoms of HES*** |  |  |  |  |  |  |  |  |
| Fatigue/tiredness | n | 35 | 5 | 9 | 3 | 0 | 0 | 52 |
|  | % | 67 | 10 | 17 | 6 | 0 | 0 |  |
| Swelling | n | 6 | 8 | 6 | 2 | 1 | 2 | 25 |
|  | % | 24 | 32 | 24 | 8 | 4 | 8 |  |
| General feeling of discomfort | n | 21 | 14 | 3 | 5 | 3 | 0 | 46 |
|  | % | 56 | 30 | 7 | 11 | 7 | 0 |  |
| Fever | n | 2 | 19 | 9 | 6 | 1 | 0 | 37 |
|  | % | 5 | 51 | 24 | 16 | 3 | 0 |  |
| Weakness | n | 16 | 13 | 8 | 0 | 0 | 0 | 37 |
|  | % | 43 | 35 | 22 | 0 | 0 | 0 |  |
| Lymph node enlargement | n | 2 | 2 | 5 | 0 | 1 | 1 | 11 |
|  | % | 18 | 18 | 45 | 0 | 9 | 9 |  |
| Chills or sweats | n | 10 | 8 | 5 | 1 | 0 | 2 | 26 |
|  | % | 38 | 31 | 19 | 4 | 0 | 8 |  |
| Weight loss | n | 4 | 0 | 8 | 4 | 2 | 8 | 26 |
|  | % | 15 | 0 | 31 | 15 | 8 | 31 |  |
| Weight gain | n | 4 | 2 | 2 | 4 | 1 | 1 | 14 |
|  | % | 29 | 14 | 14 | 29 | 7 | 7 |  |
| Eye redness | n | 6 | 2 | 2 | 0 | 0 | 1 | 11 |
|  | % | 55 | 18 | 18 | 0 | 0 | 9 |  |
| Ear pain | n | 2 | 4 | 1 | 0 | 0 | 0 | 7 |
|  | % | 29 | 57 | 14 | 0 | 0 | 0 |  |

| Cold/numbness in fingers  and/or toes | n | 12 | 4 | 2 | 0 | 1 | 1 | 20 |
| --- | --- | --- | --- | --- | --- | --- | --- | --- |
|  | % | 60 | 20 | 10 | 0 | 5 | 5 |  |

| ***Integumentary system – symptoms of HES*** |
| --- |

| Rash | n | 8 | 8 | 22 | 4 | 0 | 1 | 44 |
| --- | --- | --- | --- | --- | --- | --- | --- | --- |
|  | % | 19 | 19 | 51 | 9 | 0 | 2 |  |
| Hives | n | 4 | 5 | 9 | 3 | 0 | 0 | 21 |
|  | % | 19 | 24 | 43 | 14 | 0 | 0 |  |
| Patches of scaly skin | n | 5 | 4 | 11 | 0 | 0 | 0 | 20 |
|  | % | 25 | 20 | 55 | 0 | 0 | 0 |  |
| Itching | n | 12 | 4 | 16 | 1 | 0 | 1 | 34 |
|  | % | 35 | 12 | 47 | 3 | 0 | 3 |  |
| Tightness of the skin | n | 4 | 3 | 4 | 2 | 0 | 0 | 13 |
|  | % | 31 | 23 | 31 | 15 | 0 | 0 |  |
| Redness of the skin | n | 10 | 5 | 11 | 0 | 0 | 1 | 27 |
|  | % | 37 | 19 | 41 | 0 | 0 | 4 |  |
| Hair loss | n | 8 | 1 | 1 | 0 | 0 | 5 | 15 |
|  | % | 53 | 7 | 7 | 0 | 0 | 33 |  |
| Ulcers (e.g., mouth) | n | 2 | 2 | 5 | 3 | 0 | 1 | 13 |
|  | % | 15 | 15 | 38 | 23 | 0 | 8 |  |
| Blisters | n | 1 | 1 | 0 | 1 | 0 | 0 | 3 |
|  | % | 33 | 33 | 0 | 33 | 0 | 0 |  |
| Other^†^ | n | 0 | 0 | 1 | 2 | 1 | 0 | 4 |
|  | % | 0 | 0 | 25 | 50 | 25 | 0 |  |

| ***Respiratory system – symptoms of HES*** |
| --- |

| Wheezing | n | 9 | 9 | 20 | 4 | 1 | 1 | 44 |
| --- | --- | --- | --- | --- | --- | --- | --- | --- |
|  | % | 20 | 20 | 45 | 9 | 2 | 2 |  |
| Shortness of breath | n | 17 | 15 | 1 | 4 | 1 | 1 | 39 |
|  | % | 44 | 38 | 3 | 10 | 3 | 3 |  |
| Dry cough | n | 19 | 20 | 2 | 0 | 0 | 0 | 41 |
|  | % | 46 | 49 | 5 | 0 | 0 | 0 |  |
| Wet cough | n | 3 | 4 | 8 | 0 | 0 | 1 | 16 |
|  | % | 19 | 25 | 50 | 0 | 0 | 6 |  |
| Chest tightness | n | 9 | 19 | 4 | 1 | 1 | 0 | 34 |
|  | % | 26 | 56 | 12 | 3 | 3 | 0 |  |
| Chest pain/discomfort | n | 9 | 17 | 3 | 2 | 0 | 1 | 32 |
|  | % | 28 | 53 | 9 | 6 | 0 | 3 |  |
| Sinus pressure or discomfort | n | 14 | 11 | 4 | 2 | 1 | 0 | 32 |
|  | % | 44 | 34 | 13 | 6 | 3 | 0 |  |
| Stuffy nose | n | 13 | 16 | 2 | 1 | 1 | 0 | 33 |
|  | % | 39 | 48 | 6 | 3 | 3 | 0 |  |
| Sore throat | n | 4 | 12 | 10 | 3 | 1 | 0 | 30 |
|  | % | 13 | 40 | 33 | 10 | 3 | 0 |  |
| Inability or decreased  sense of smell | n | 7 | 7 | 3 | 2 | 0 | 1 | 20 |
|  | % | 35 | 35 | 15 | 10 | 0 | 5 |  |
| Postnasal drip | n | 9 | 5 | 4 | 0 | 0 | 0 | 18 |
|  | % | 50 | 28 | 22 | 0 | 0 | 0 |  |
| Runny nose | n | 9 | 9 | 2 | 1 | 0 | 0 | 21 |
|  | % | 43 | 43 | 10 | 5 | 0 | 0 |  |

| ***Nervous system – symptoms of HES*** | | |  |  |  |  |  |  |
| --- | --- | --- | --- | --- | --- | --- | --- | --- |
| Headache | n | 6 | 11 | 6 | 0 | 0 | 0 | 23 |
|  | % | 26 | 48 | 26 | 0 | 0 | 0 |  |
| Dizziness | n | 7 | 9 | 3 | 1 | 1 | 0 | 21 |
|  | % | 33 | 43 | 14 | 5 | 5 | 0 |  |
| Tightness in extremities | n | 3 | 4 | 2 | 1 | 1 | 0 | 11 |
|  | % | 27 | 36 | 18 | 9 | 9 | 0 |  |
| Numbness in hands or feet | n | 10 | 7 | 0 | 0 | 0 | 0 | 17 |
|  | % | 59 | 41 | 0 | 0 | 0 | 0 |  |
| Loss of motor control or falling | n | 1 | 6 | 1 | 2 | 0 | 1 | 11 |
|  | % | 9 | 55 | 9 | 18 | 0 | 9 |  |
| Weakness in arms or legs | n | 7 | 5 | 2 | 0 | 0 | 0 | 14 |
|  | % | 50 | 36 | 14 | 0 | 0 | 0 |  |
| Changes in behavior | n | 4 | 1 | 2 | 0 | 0 | 1 | 8 |
|  | % | 50 | 13 | 25 | 0 | 0 | 13 |  |
| Difficulty concentrating | n | 8 | 5 | 1 | 2 | 0 | 0 | 16 |
|  | % | 50 | 31 | 6 | 13 | 0 | 0 |  |
| Memory problems | n | 7 | 1 | 5 | 0 | 0 | 1 | 14 |
|  | % | 50 | 7 | 36 | 0 | 0 | 7 |  |
| Problems with speech | n | 3 | 2 | 3 | 1 | 0 | 0 | 9 |
|  | % | 33 | 22 | 33 | 11 | 0 | 0 |  |
| Problems with vision | n | 6 | 2 | 1 | 0 | 0 | 1 | 10 |
|  | % | 60 | 20 | 10 | 0 | 0 | 10 |  |
| Other^‡^ | n | 1 | 0 | 0 | 0 | 0 | 0 | 1 |
|  | % | 100 | 0 | 0 | 0 | 0 | 0 |  |

| ***Muscular system – symptoms of HES*** | | | | | | | | |
| --- | --- | --- | --- | --- | --- | --- | --- | --- |
| Muscle spasms | n | 6 | 7 | 4 | 0 | 0 | 0 | 17 |
|  | % | 35 | 41 | 24 | 0 | 0 | 0 |  |
| Muscle pain | n | 10 | 7 | 14 | 1 | 0 | 2 | 34 |
|  | % | 29 | 21 | 41 | 3 | 0 | 6 |  |
| Joint pain | n | 15 | 6 | 9 | 1 | 0 | 1 | 32 |
|  | % | 47 | 19 | 28 | 3 | 0 | 3 |  |
| Joint swelling | n | 4/26) | 2 | 6 | 2 | 0 | 1 | 16 |
|  | % | 31 | 13 | 38 | 13 | 0 | 6 |  |
| ***Digestive system – symptoms of HES*** | | | | | | | | |
| Nausea | n | 7 | 11 | 15 | 1 | 0 | 0 | 34 |
|  | % | 21 | 32 | 44 | 3 | 0 | 0 |  |
| Vomiting | n | 1 | 9 | 13 | 3 | 0 | 1 | 27 |
|  | % | 4 | 33 | 48 | 11 | 0 | 4 |  |
| Lack of appetite | n | 13 | 12 | 2 | 1 | 0 | 0 | 28 |
|  | % | 46 | 43 | 7 | 4 | 0 | 0 |  |
| Difficulty swallowing or food  getting stuck | n | 7 | 20 | 1 | 1 | 0 | 0 | 29 |
|  | % | 24 | 69 | 3 | 3 | 0 | 0 |  |
| Diarrhea | n | 7 | 9 | 15 | 1 | 0 | 0 | 32 |
|  | % | 22 | 28 | 47 | 3 | 0 | 0 |  |
| Pain in the abdomen | n | 10 | 8 | 10 | 1 | 0 | 0 | 29 |
|  | % | 34 | 28 | 34 | 3 | 0 | 0 |  |
| Constipation | n | 1 | 2 | 5 | 1 | 0 | 1 | 10 |
|  | % | 10 | 20 | 50 | 10 | 0 | 10 |  |
| Yellow skin/jaundice | n | 0 | 0 | 1 | 0 | 1 | 0 | 2 |
|  | % | 0 | 0 | 50 | 0 | 50 | 0 |  |

| ***Circulatory system – symptoms of HES*** |
| --- |

| Chest pain | n | 1 | 6 | 2 | 1 | 0 | 0 | 10 |
| --- | --- | --- | --- | --- | --- | --- | --- | --- |
|  | % | 10 | 60 | 20 | 10 | 0 | 0 |  |
| Swelling in legs | n | 2 | 1 | 0 | 0 | 0 | 0 | 3 |
|  | % | 67 | 33 | 0 | 0 | 0 | 0 |  |
| Heart palpitations/irregular  heartbeat | n | 3 | 5 | 3 | 1 | 0 | 0 | 12 |
|  | % | 25 | 42 | 25 | 8 | 0 | 0 |  |
| Shortness of breath | n | 6 | 3 | 2 | 0 | 0 | 0 | 11 |
|  | % | 55 | 27 | 18 | 0 | 0 | 0 |  |
| Dizziness | n | 6 | 2 | 2 | 1 | 0 | 0 | 11 |
|  | % | 55 | 18 | 18 | 9 | 0 | 0 |  |
| Throat/jaw pain | n | 1 | 3 | 4 | 0 | 0 | 0 | 8 |
|  | % | 13 | 38 | 50 | 0 | 0 | 0 |  |
| Sweating | n | 5 | 3 | 0 | 1 | 0 | 0 | 9 |
|  | % | 56 | 33 | 0 | 11 | 0 | 0 |  |

Includes responses to items 37, 41, 45, 54, 58, 64, 68, and 74 of the survey instruments (**Table E1**).
*Non-mutually exclusive groups. ^†^Includes bruising, subcutaneous nodules, thickening of skin, decreased sensation, eczema. ^‡^Includes fainting, excessive sleep, mixed axonal and generalized demyelinating, sensorimotor polyneuropathy, jerky movements, tingling sensation in fingers and toes.

Abbreviations: HES, hypereosinophilic syndromes; N/A, not applicable.

### **Table E7.** HCRU (past 12 months)

| **HES HCRU over past 12 months** | **All respondents (N=54)** | |
| --- | --- | --- |
| **Urgent care (N=3)** | **N** | **%** |
| Not at all | 0 | 0 |
| 1–3 times | 1 | 33 |
| 4–6 times | 2 | 67 |
| 7–9 times | 0 | 0 |
| ≥10 times | 0 | 0 |
| **Emergency room (N=11)** | **N** | **%** |
| Not at all | 2 | 18 |
| 1–3 times | 7 | 64 |
| 4–6 times | 1 | 9 |
| 7–9 times | 1 | 9 |
| ≥10 times | 0 | 0 |
| **Hospital admission (N=27)** | **N** | **%** |
| Not at all | 0 | 0 |
| 1–3 times | 26 | 96 |
| 4–6 times | 0 | 0 |
| 7–9 times | 1 | 4 |
| ≥10 times | 0 | 0 |
| **Primary care provider (N=28)** | **N** | **%** |
| Not at all | 1 | 4 |
| 1–3 times | 17 | 61 |
| 4–6 times | 7 | 25 |
| 7–9 times | 1 | 4 |
| ≥10 times | 2 | 7 |
| **Allergist/immunologist (N=27)** | **N** | **%** |
| Not at all | 1 | 4 |
| 1–3 times | 13 | 48 |
| 4–6 times | 10 | 37 |
| 7–9 times | 1 | 4 |
| ≥10 times | 2 | 7 |
| **Pulmonologist (N=17)** | **N** | **%** |
| Not at all | 1 | 6 |
| 1–3 times | 14 | 82 |
| 4–6 times | 2 | 12 |
| 7–9 times | 0 | 0 |
| ≥10 times | 0 | 0 |
| **Other specialist*** **(N=15)** | **N** | **%** |
| Not at all | 0 | 0 |
| 1–3 times | 11 | 73 |
| 4–6 times | 3 | 20 |
| 7–9 times | 1 | 7 |
| ≥10 times | 0 | 0 |

Includes responses to item 101 of the survey instrument (**Table E1**).
*Includes gastroenterologists, ophthalmologists, hematologists, oncologists, and NIH (n=1 patient report in current study).
Abbreviations: HCRU, healthcare resource utilization; HES, hypereosinophilic syndromes; NIH, National Institute of Health.

### **Table E8**. Time to HES treatment initiation and impact of treatment for HES

| **Time from HES diagnosis to treatment initiation** | **n** | **%** |
| --- | --- | --- |
| <3 months | 30 | 56 |
| 3–6 months | 12 | 22 |
| 6 months–1 year | 5 | 9 |
| 1–3 years | 3 | 6 |
| 3–5 years | 3 | 6 |
| 5–7 years | 0 | 0 |
| 7–10 years | 0 | 0 |
| >10 years | 1 | 2 |
| **"I feel that taking care of my HES (e.g., treatment time, rest and recovery, visits with HCPs) takes time away from other activities."** | **n** | **%** |
| Strongly disagree | 0 | 0 |
| Somewhat disagree | 3 | 6 |
| Neither agree nor disagree | 14 | 26 |
| Somewhat agree | 20 | 37 |
| Strongly agree | 16 | 30 |
| N/A or prefer not to answer | 14 | 26 |

Includes responses to items 26 and 34 of the survey instrument (**Table E1**).

Abbreviations: HCP, healthcare provider; HES, hypereosinophilic syndromes; N/A, not applicable.

### **Table E9**. HES treatment burden

| **HES treatment impact^*^** | | **Strongly disagree** | **Somewhat disagree** | **Neither agree nor disagree** | **Somewhat agree** | **Strongly agree** | **N/A or prefer  not to answer** |
| --- | --- | --- | --- | --- | --- | --- | --- |
|  | **N** | **n (%)** | | | | | |
| **This treatment helped/helps control my HES symptoms** | | | | | | | |
| Steroids (prednisone/methylprednisolone)^†^ | 39 | 1 (3) | 2 (5) | 3 (8) | 14 (36) | 19 (49) | 0 (0) |
| Hydroxyurea | 28 | 3 (11) | 3 (11) | 7 (25) | 8 (29) | 7 (25) | 0 (0) |
| Chlorambucil | 18 | 0 (0) | 1 (6) | 4 (22) | 8 (44) | 5 (28) | 0 (0) |
| Vincristine | 12 | 0 (0) | 0 (0) | 1 (8) | 4 (33) | 7 (58) | 0 (0) |
| Methotrexate | 9 | 3 (33) | 0 (0) | 0 (0) | 4 (44) | 2 (22) | 0 (0) |
| Cyclosporine | 11 | 0 (0) | 5 (45) | 3 (27) | 3 (27) | 0 (0) | 0 (0) |
| Azathioprine | 1 | 0 (0) | 1 (100) | 0 (0) | 0 (0) | 0 (0) | 0 (0) |
| Ivermectin | 4 | 0 (0) | 0 (0) | 1 (25) | 2 (50) | 0 (0) | 1 (25) |
| Tyrosine kinase inhibitors | 5 | 2 (40) | 1 (20) | 1 (20) | 1 (20) | 0 (0) | 0 (0) |
| Monoclonal antibody injectable medications^‡^ | 25 | 0 (0) | 3 (12) | 2 (8) | 5 (20) | 15 (60) | 0 (0) |
| Non-prescription management^§^ | 13 | 2 (15) | 2 (15) | 5 (38) | 2 (15) | 1 (8) | 1 (7.69) |
| Interferon alpha | 6 | 1 (17) | 2 (33) | 1 (17) | 0 (0) | 2 (33) | 0 (0) |
| **I am satisfied with how this treatment controlled/controls my HES symptoms** | | | | | | | |
| Steroids (prednisone/methylprednisolone)^†^ | 38 | 7 (18) | 2 (5) | 2 (5) | 17 (45) | 9 (24) | 1 (3) |
| Hydroxyurea | 28 | 2 (7) | 2 (7) | 5 (18) | 15 (54) | 4 (14) | 0 (0) |
| Chlorambucil | 18 | 0 (0) | 3 (17) | 4 (22) | 8 (44) | 3 (17) | 0 (0) |
| Vincristine | 12 | 0 (0) | 0 (0) | 3 (25) | 5 (42) | 4 (33) | 0 (0) |
| Methotrexate | 9 | 3 (33) | 0 (0) | 2 (22) | 4 (44) | 0 (0) | 0 (0) |
| Cyclosporine | 11 | 0 (0) | 3 (27) | 5 (45) | 3 (27) | 0 (0) | 0 (0) |
| Azathioprine | 1 | 0 (0) | 1 (100) | 0 (0) | 0 (0) | 0 (0) | 0 (0) |
| Ivermectin | 4 | 1 (25) | 0 (0) | 0 (0) | 2 (50) | 0 (0) | 1 (25) |
| Tyrosine kinase inhibitors | 5 | 1 (20) | 2 (40) | 2 (40) | 0 (0) | 0 (0) | 0 (0) |
| Monoclonal antibody injectable medications^‡^ | 25 | 1 (4) | 2 (8) | 4 (16) | 3 (12) | 14 (56) | 1 (4) |
| Non-prescription management^§^ | 13 | 3 (23) | 2 (15) | 3 (23) | 2 (15) | 2 (15) | 1 (8) |
| Interferon alpha | 6 | 2 (33) | 0 (0) | 1 (17) | 1 (17) | 2 (33) | 0 (0) |
| **I feel like this treatment helped/helps improve my quality of life** | | | | | | | |
| Steroids (prednisone/methylprednisolone)^†^ | 40 | 9 (23) | 5 (13) | 5 (13) | 12 (30) | 8 (20) | 1 (3) |
| Hydroxyurea | 28 | 3 (11) | 3 (11) | 7 (25) | 9 (32) | 6 (21) | 0 (0) |
| Chlorambucil | 18 | 0 (0) | 2 (11) | 5 (28) | 6 (33) | 5 (28) | 0 (0) |
| Vincristine | 12 | 0 (0) | 1 (8) | 4 (33) | 6 (50) | 1 (8) | 0 (0) |
| Methotrexate | 9 | 3 (33) | 0 (0) | 1 (11) | 3 (33) | 2 (22) | 0 (0) |
| Cyclosporine | 11 | 0 (0) | 3 (27) | 2 (18) | 5 (45) | 1 (9) | 0 (0) |
| Azathioprine | 1 | 0 (0) | 1 (100) | 0 (0) | 0 (0) | 0 (0) | 0 (0) |
| Ivermectin | 4 | 1 (25) | 0 (0) | 1 (25) | 1 (25) | 0 (0) | 1 (25) |
| Tyrosine kinase inhibitors | 5 | 2 (40) | 1 (20) | 1 (20) | 1 (20) | 0 (0) | 0 (0) |
| Monoclonal antibody injectable medications^‡^ | 25 | 1 (4) | 2 (8) | 3 (12) | 4 (16) | 14 (56) | 1 (4) |
| Non-prescription management^§^ | 13 | 3 (23) | 2 (15) | 4 (31) | 1 (8) | 2 (15) | 1 (8) |
| Interferon alpha | 6 | 2 (33) | 0 (0) | 1 (17) | 2 (33) | 1 (17) | 0 (0) |
| **In general, this treatment is/was overall easy to manage (i.e., ease of administration, storage, etc.)** | | | | | | | |
| Steroids (prednisone/methylprednisolone)^†^ | 40 | 1 (3) | 3 (8) | 9 (23) | 14 (35) | 12 (30) | 1 (3) |
| Hydroxyurea | 28 | 0 (0) | 0 (0) | 6 (21) | 17 (61) | 5 (18) | 0 (0) |
| Chlorambucil | 18 | 0 (0) | 2 (11) | 8 (44) | 7 (39) | 1 (6) | 0 (0) |
| Vincristine | 12 | 0 (0) | 0 (0) | 8 (67) | 4 (33) | 0 (0) | 0 (0) |
| Methotrexate | 9 | 0 (0) | 0 (0) | 1 (11) | 5 (56) | 3 (33) | 0 (0) |
| Cyclosporine | 11 | 0 (0) | 2 (18) | 2 (18) | 4 (36) | 3 (27) | 0 (0) |
| Azathioprine | 1 | 0 (0) | 0 (0) | 0 (0) | 0 (0) | 1 (100) | 0 (0) |
| Ivermectin | 4 | 0 (0) | 0 (0) | 1 (25) | 1 (25) | 1 (25) | 1 (25) |
| Tyrosine kinase inhibitors | 5 | 0 (0) | 0 (0) | 2 (40) | 1 (20) | 2 (40) | 0 (0) |
| Monoclonal antibody injectable medications^‡^ | 25 | 0 (0) | 3 (12) | 2 (8) | 11 (44) | 8 (32) | 1 (4) |
| Non-prescription management^§^ | 13 | 1 (8) | 0 (0) | 4 (31) | 1 (8) | 6 (46) | 1 (8) |
| Interferon alpha | 6 | 0 (0) | 1 (17) | 3 (50) | 1 (17) | 1 (17) | 0 (0) |

Includes responses to items 29–32 of the survey instrument (**Table E1**).
*Non-mutually exclusive groups. ^†^Administration route not specified. ^‡^Includes mepolizumab, alemtuzumab, and benralizumab. ^§^Includes OTC medications, Tylenol, Flonase nasal spray.

Abbreviations: HES, hypereosinophilic syndromes; OTC, over-the-counter.

### **Table E10**. The patient voice in HES

| ***US RESPONDENTS – Item 99 – What is one thing you want your doctor to know about your HES?*** |
| --- |
| Pain is real |
| It’s debilitating mentally and physically |
| Please cure us! |
| Please start treatment asap |
| How hard it is mentally knowing your dying |
| There is no cure but that does not mean there is no hope. It is not in “my head” these symptoms are real and I deserve your time and attention. |
| It is real |
| Do not want side effects of drugs |
| It's real |
| That a lot of the symptoms I experience such as many of those on this survey are related to my HES and not something else. He always wants to look for other things that might be going on and when he can't find other reasons then says it may be HES relate. I'm sure they are HES related |
| Very frustrating |
| It affects my entire body in one way or another and it is real & relentless and disabling |
| My providers are wonderful - I have no concerns there |
| The lack of compassion and understanding |
| That I actually have been downplaying my pain & symptoms. However, when I do tell them something it is true. I have explained for years that my disease was deeper (than GI) and in other places; however, they blew me off for decades. I now have developed MGUS and I and some docs believe that chronic inflammation contributed. I also have Chronic Pancreatitis which increases my risk for Pancreatic Cancer. The Chronic Inflammation also caused severe arterial calcification from damage to the arteries. I was diagnosed with MS; however, it looks like the damage (Microvascular Ischemia) is really from EGPA/HES.  My disease has been killing me & I am sure it will take years off my life. I have suffered untreated for decades! |
| Early signs of it so it can be diagnosed correctly so no one else has to have cardiac failure to figure it out. Also, that we want more treatment options. Prednisone is not a long-term care option |
| I’m frustrated |
| Truly know how it impacts my life |
| I am always in pain |
| I've told them all -- the ones that have helped know why and how they have. I tell them the importance of them asking open-ended questions and really LISTENING to the answers. I also tell them how important I found it to have my diagnosing doctor (who is no longer practicing in the area) be willing to push back against other physicians who wanted to just write me and my symptoms off. The ones that haven't helped know how they screwed up and how it cost me (and not one of them as expressed any remorse/sadness etc., even though it is many years past any potential for lawsuits -- and I don't believe in filing many suits). But I've also told them just how their actions and specific practices cost me/hurt me -- and what they should have done differently |
| Talk about how it can progress. What if my medicine doesn’t work anymore. |
| I don't feel as normal/healthy as I look and many things are much harder than before |
| Why can’t medical science do something about this disease.? Or identify what triggers it. Every minute you feel different. It’s a terrible way to live |
| Please don't treat me like an idiot. I know my body and how it feels - learn about HES and be present and helpful not just treat symptoms as they arise |
| It’s tiring and I worry it could get worse |
| Long-term testing and re-evaluation best practices |
| ***US RESPONDENTS – Item 104 – Anything else you would like to tell us about your HES?*** |
| None |
| Thank you for caring! |
| I'm really in financial trouble and mental illness |
| I felt that life was meaningless |
| I had high numbers for 10 yrs before I had any kind of treatment and I believe that allowed the symptoms to worsen. Since I have been on chemo treatments, we are only seeing "allergy" level eosinophils at this time |
| Help me please |
| I could write a book! But this survey was long enough… |
| I'm desperate |
| None |
| None |
| No |
| N/A |
| This has bothered me all my life |
| As I understand it, HES is a condition that is a component of my other illnesses (or cause?), and there isn't a lot known about the work of eosinophils in the body. I don't think providers keep an open mind about how the disease involves the entire body and yet can strike any one part of it severely and without notice. I suspect my bone issues are related for instance but I don't know. It's just something I'd like providers to be aware of. THEY NEED TO TALK TO PATIENTS MORE and LET US KNOW WHAT'S GOING ON |
| N/A |
| No, thanks |
| None |
| Hopeless |
| It is very difficult to find a doctor that is knowledgeable about HES, how to diagnose it, and how to treat it |
| No, thanks |
| No |
| This world is unfair to me |
| Very frustrating not knowing what the cause is at this time |
| It sucks :) |
| Because of the diuretic dosage needed to control my CHF, I developed gout and chronic kidney disease. Because of the prednisone, I now have bone density concerns. HES changed my life significantly |
| This took me at least 2 to 3 hours |
| My allergist and I wonder if my HES was symptomatic of my growing breast cancer. I was not diagnosed with that for 2 more years. Could they be related? |
| My HES has resulted in debilitating Chronic Pain |
| This disease is trashing my body |
| If I had known about the disease in the early '90s, I would have recognized the symptoms as long-standing and might have been able to fight against all the doctors who jumped to easy misdiagnoses and the concurrent mis-treatments, and all the damage that arose because of them (like the extreme use of steroids all the time, which ultimately caused a number of chronic problems now). 30 years of doctors telling me that I needed to "expect changes" at my age, "accept these issues come with menopause even early," and my personal favorite, "you need more sex -- that will take care of these problems." I used to think there must be something wrong with women who complained about their doctors not listening to them, or thinking every problem could be cured with an antidepressant or two ... or three (what mine tried, which finally made me realize there was a problem with my old PCP). Now I tell doctors that they need to ask open-ended questions and listen to the answers. If they don't have time, have someone on their staffs trained to do so, and listen to the answers without bias toward the "usual" explanation. And I tell patients if their doctors DON'T do that, to thank them for their time, go out, wait for a copy of their records -- including any labs -- and then go find another doctor |
| Some people say you need monthly blood work to check for cancer. I had one bone marrow biopsy but nothing after that. I do blood work at allergist every quarter |
| This is a debilitating disease. I wish more doctors knew about it. Having to travel to see someone is brutal. Many times, I find a doctor posts on his website he treats HES or other Eosinophilia disease but after I travel to get there I find they don’t know about it at all. It’s like click bait, false hope. It’s discouraging |
| I wish more doctors knew about this disease. I really felt like I was in good hands with Dr. X when she was practicing. I would drive for hours to see her several times a year but always was taken well care of. This disease is progressing and I know my body is slowing down and eventually I will succumb to it. I take day by day because I know I have to have QUALITY over quantity of life |
| I would just like a cure |
| One of the biggest struggles is provider knowledge of HES and related eosinophilic disorders, and the necessity of coordinated care between difference specialties. I feel that I am the primary driver of my care and treatment via educating myself on HES via medical journals on differential diagnosis, treatment, and interdisciplinary care |
