# Supplementary material for: Burden of hypereosinophilic syndromes in the United States: Patients’ perspective
Source: J Allergy Clin Immunol Glob. 2025 May 28;4(3):100501. doi: 10.1016/j.jacig.2025.100501 (PMC12246599; doi:10.1016/j.jacig.2025.100501)
Supplement: Supplementary Data [file mmc2.docx]

# **eSupplement Figure Legends**

# **Figure E1.** Frequency of adverse impacts of HES on QoL including daily activities, work/school activities, social life/relationship activities, and other activities

Figure E1 reports the results for items 83 (Daily activities); 86 (Work/school activities); 89 (Social life/relationship activities); and /92 (Other activities) of the survey instrument (**Table E1**).
*Also includes response ‘Prefer not to answer’. Groups are non-mutually exclusive.
Abbreviations: HES, hypereosinophilic syndromes; QoL, quality of life.

**Figure E2**. Word cloud representation of US patients with HES and caregiver responses to the question: ‘What is one thing you want your doctor to know about your HES?’
Abbreviation: HES, hypereosinophilic syndromes.
