# Supplementary figures and images for: Burden of hypereosinophilic syndromes in the United States: Patients’ perspective
Source: J Allergy Clin Immunol Glob. 2025 May 28;4(3):100501. doi: 10.1016/j.jacig.2025.100501 (PMC12246599; doi:10.1016/j.jacig.2025.100501)

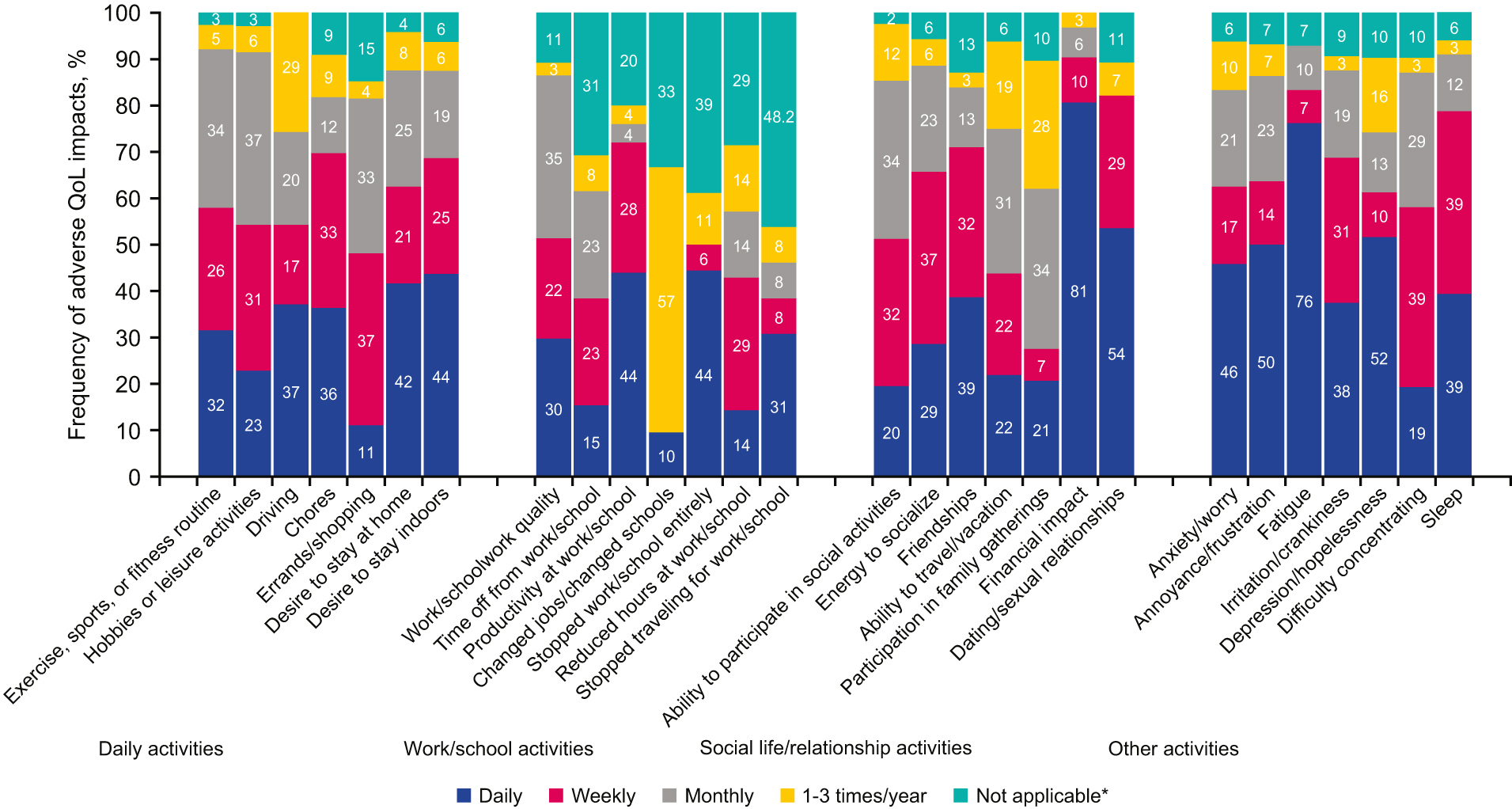

Supplement: Supplementary Fig 1 [file figs1.jpg]

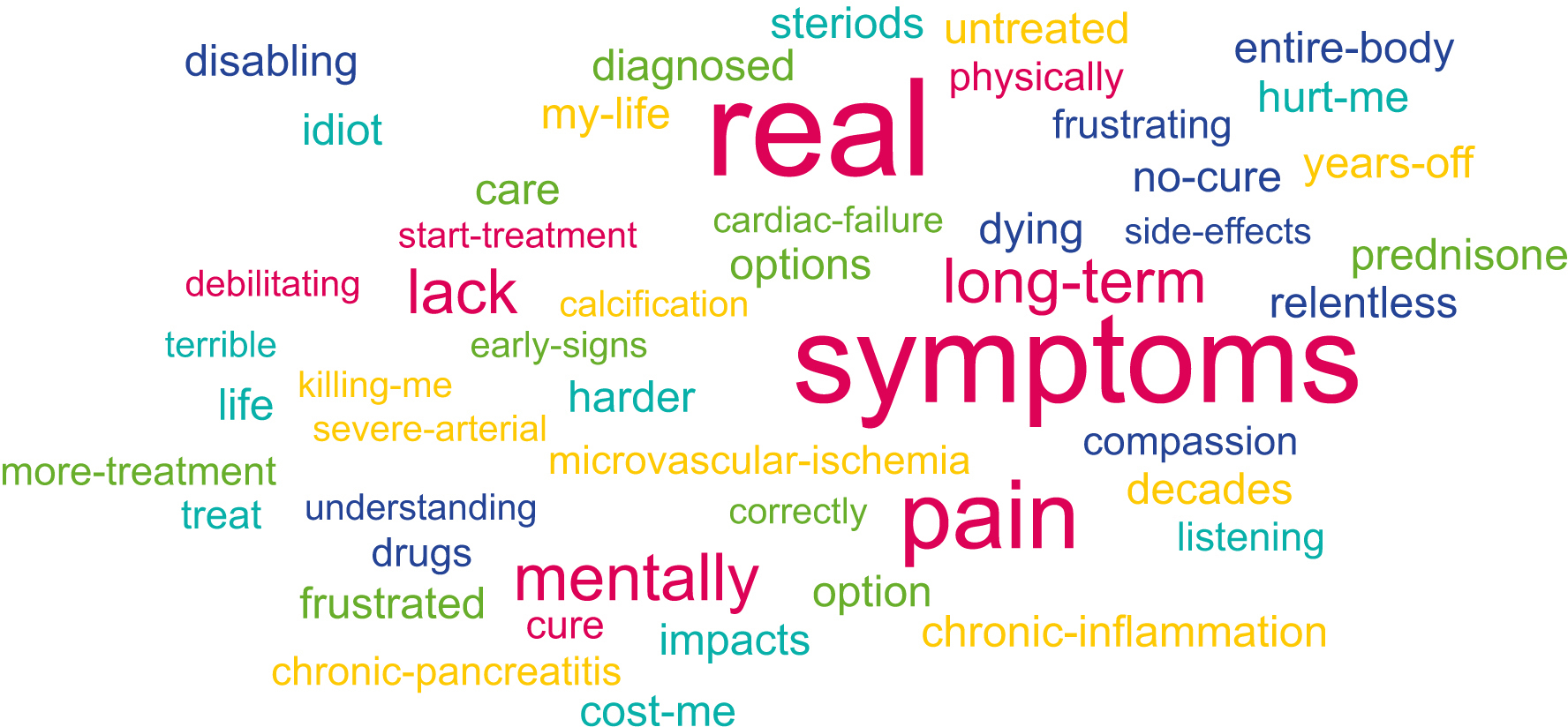

Supplement: Supplementary Fig 2 [file figs2.jpg]
